# Hierarchical MXene/ZnO Nanorods: WO_3_/CNT Trilayer Coatings on Cotton for High‐Performance Multifunctional Wearable Fabrics

**DOI:** 10.1002/advs.202521175

**Published:** 2026-03-18

**Authors:** Thirumalaisamy Suryaprabha, Chunghyeon Choi, Sujith Lal, Min‐Young Seo, Tae‐Wook Kim, Ergang Wang, Byungil Hwang

**Affiliations:** ^1^ Department of Intelligent Semiconductor Engineering Chung‐Ang University Seoul Republic of Korea; ^2^ School of Integrative Engineering Chung‐Ang University Seoul Republic of Korea; ^3^ Department of Flexible and Printable Electronics, LANL‐JBNU Engineering Institute‐Korea Jeonbuk National University Jeonju Republic of Korea; ^4^ Department of JBNU‐KIST Industry‐Academia Convergence Research Jeonbuk National University Jeonju Republic of Korea; ^5^ Department of Chemistry and Chemical Engineering Chalmers University of Technology Göteborg Sweden

**Keywords:** EMI shielding, multifunctional cotton, MXene, strain sensor, wearable electronics

## Abstract

Fabric‐based wearable electronics are gaining increasing attention owing to their flexibility, breathability, biocompatibility, and seamless integration into clothing. However, most existing studies rely primarily on metallic or carbon‐based conductive materials. In contrast, the integration of semiconducting metal oxides in wearable textiles remains limited, despite their advantages in achieving tunable electrical and thermal responses. In this study, we developed a sandwich‐structured coating on cotton fabric, where a semiconductive layer of WO_3_‐doped ZnO nanorods was embedded between two conductive layers of MXene and carbon nanotubes (CNTs). This hierarchical and heterogeneous coating architecture enabled synergistic interactions that significantly enhance multifunctional performance. The engineered fabric exhibited reliable strain sensing with a short response and recovery time (∼200 ms), excellent mechanical durability over 2000 stretch/release cycles, and the ability to monitor human motion. Furthermore, the fabric demonstrated efficient Joule heating, reaching ∼110°C within ∼15 s, and high electromagnetic interference (EMI) shielding effectiveness (∼34.4 dB), which increased to ∼78 dB by raising fabric thickness, meeting commercial EMI standards. Notably, these functionalities were achieved without compromising flexibility, light weight, and breathability. Thus, this study presents a new paradigm for designing multifunctional textile electronics by integrating semiconductive and conductive nanomaterials, overcoming the limitations of conventional conductive‐only approaches.

## Graphical abstract

xx xx.

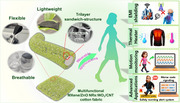

## 1 Introduction

Rapid advances in technology and the growing emphasis on sustainability have ttransformed the traditional utility of textiles from simple, protective materials into smart, interactive platforms [1, 2]. In this context, multifunctional textiles that integrate two or more advanced features such as flexible sensing, real‐time health monitoring, energy harvesting and storage, electromagnetic interference (EMI) shielding, thermal regulation, water resistance, and antibacterial protection are emerging as the foundations for the next‐generation wearable technologies [3, 4, 5, 6]. These multifunctional fabrics deliver advanced performances without compromising breathability, flexibility, or user comfort, while reducing material use and environmental impact by combining multiple functions into a single, sustainable system.

Furthermore, to impart multiple functionalities within a single textile, recent strategies have increasingly focused on synergistic combinations of electrically conductive nanomaterials, wherein materials with complementary properties are integrated to achieve enhanced or emergent performance. For example, Zhang et al. [7] employed a MXene/carbon nanotube (CNT) hybrid coating on fabric, demonstrating superior EMI shielding effectiveness (EMI SE), efficient Joule heating capability, and high strain‐sensing sensitivity. Liu et al. [8] utilized a polypyrrole/silver nanowire (Ag NWs) coating on textile substrates for EMI shielding, while also achieving integrated functionalities such as Joule heating and antibacterial activity. This hybrid coatings exhibited excellent EMI shielding and joule heating performance. In another study, Yi et al. [9] employed MXene nanosheets to improve the adhesion of liquid metal (LM) droplets to textile surfaces and to form continuous conductive pathways between adjacent droplets. The resulting multifunctional textiles exhibited excellent chemical resistance and thermal stability, making them suitable for robust wearable applications. Furthermore, Abay et al. [10] developed MXene/graphene oxide (GO)/AgNW hybrid electronic textiles that not only demonstrated high EMI shielding efficiency but also delivered outstanding pressure‐sensing performance.

Despite their promising multifunctionality, synergistic conductive nanomaterials face key limitations. A major drawback of MXene‐based systems is their inherent tendency to restack due to van der Waals interactions, which significantly reduces active surface area and limits ion transport both critical factors in high‐performance sensing, energy storage, and thermal management applications [11, 12, 13]. Moreover, the integration of nanostructured conductive fillers (such as GO, CNTs, AgNW or MXene) with conductive polymers often suffers from limited interfacial compatibility, leading to poor dispersion and, consequently, compromised electrical conductivity and mechanical stability [14, 15, 16,65, 66]. Recent research on LMs‐based hybrid coatings for multifunctional textile applications has shown promising results in terms of conductivity and stretchability. However, these systems often require high LM loading to form effective percolation networks, increasing the risk of leakage and introducing processing difficulties, particularly for wearable and thermal management applications [17, 67, 68]. To address these challenges, recent strategies have focused on incorporating additional functional materials, such as semiconductive metal oxides, alongside conductive fillers to act as interfacial bridges. This approach improves material compatibility and fosters synergistic multifunctionality within hierarchically structured multilayer architectures [18].

Zinc Oxide (ZnO) is a lightweight wide‐bandgap semiconductor that has been widely employed in functional composites owing to its favorable dielectric properties and morphological tunability into nanorods, nanowires, and hierarchical architectures [19]. In previous reports, the incorporation of ZnO into conductive composites for EMI shielding and microwave absorption has been predominantly attributed to enhanced interfacial polarization rather than intrinsic conductivity contributions [20, 21, 22, 23, 24]. Notably, interfacial polarization is not only critical for electromagnetic attenuation but also plays an important role in piezoelectric/piezoresistive behavior [25, 26], where charge accumulation and redistribution at heterogeneous interfaces govern resistance modulation under deformation. Compared with homogeneous media, heterogeneous systems exhibit stronger interfacial polarization due to differences in conductivity and permittivity across interfaces [27, 28, 29]. Accordingly, the uniform integration of ZnO within conductive MXene and CNT networks facilitates the formation of more polarization‐active interfaces. Furthermore, WO_3_ doping with the ZnO introduces additional semiconducting heterojunctions, increasing its interfacial complexity further. Herein, we engineered a ternary architecture incorporating ZnO nanorods (NRs) doped with WO_3_ as a semiconducting interlayer, sandwiched between conductive MXene and CNT layers. The novelty of this work lies in strategically positioning the ZnO NRs: WO_3_ layer to bridge and construct heterogeneous interfaces with the MXene layer and CNT network, enabling a unified textile to exhibit excellent performance across all multifunctional applications.

Together with the exceptional conductivity of MXene, the dielectric polarization and piezoelectric sensitivity of WO_3_‐doped ZnO nanorods, and the highly stretchable conductive network of CNTs, this unique hierarchical architecture delivers outstanding multifunctional performance. It achieves a gauge factor of 0.47 with ultrafast response/recovery times (∼200 ms) for strain sensing, EMI SE of 34.4 dB (scalable to 78 dB), and rapid Joule heating of 110°C within ∼15 s at 3.5 V surpassing most reported textile systems. Beyond superior metrics, the combination of fast electromechanical responsiveness, stable signal reproducibility, and mechanical robustness under cyclic loading makes this fabric particularly suitable for mission‐critical applications such as emergency Morse‐code signaling and safety‐line alert systems in industrial or hazardous environments. These capabilities ensure real‐time detection and reliable signal transmission, even under dynamic deformation or harsh operating conditions. These synergistic capabilities redefine the design framework for multifunctional wearable textiles, paving the way for their integration into next‐generation smart and adaptive systems.

The multilayer coating architecture offers a versatile and modular approach to functionalizing textile substrates, enabling the integration of diverse functional materials while maintaining structural uniformity and tunability. It leverages the distinct advantages of each layer and promotes synergistic interfacial interactions. This not only enhances coating uniformity and fabric multifunctionality but also mitigates key drawbacks of hybrid systems such as filler restacking, poor dispersion, and interfacial incompatibility by spatially separating components into well‐defined functional layers [30, 31].

## 2 Materials and Methods

### 2.1 Materials

Titanium aluminum carbide powder (Ti_3_AlC_2_, purity ≥90%, particle size ≤40 µm) was purchased from Sigma–Aldrich. Lithium fluoride (LiF, purity 99%) was obtained from FUJIFILM Wako Pure Chemical Corporation, Osaka, Japan. Hydrochloric acid (HCl, concentration 35%), zinc nitrate hexahydrate (Zn(NO_3_)_2_·6H_2_O, purity 98%), hexamethylenetetramine (purity 98.5%), and sodium tungstate dihydrate (Na_2_WO_4_·2H_2_O, purity 98%) were purchased from Daejung Chemicals, Republic of Korea. Jenotube 6A multi‐walled carbon nanotubes (MWCNTs) were sourced from JEIO Co., Ltd., Incheon, Republic of Korea. All chemicals were used as received, without further purification.

## 3 Methods

### 3.1 Synthesis of MXene

Ti_3_C_2_T_x_ MXene was synthesized from Ti_3_AlC_2_ powder using the minimally intensive layer delamination method [7]. LiF (1.6 g) was dissolved in 15.23 mL of HCl and 4.7 mL of deionized (DI) water under stirring, followed by the slow addition of Ti_3_AlC_2_ powder (2 g) to minimize heat generation from the exothermic etching reaction. Etching was conducted at 35°C for 24 h under continuous stirring at 300 rpm. The resulting suspension was washed 2–3 times with DI water by repeated centrifugation at 9000 rpm, followed by decantation. When the pH of the supernatant reached approximately 5–6, the mixture was centrifuged again at 3500 rpm for 30 min to yield a Ti_3_C_2_T_x_ aqueous dispersion with a final concentration of 10 mg/mL.

### 3.2 Synthesis of Tungsten Oxide (WO_3_)

Sodium tungstate (1.2 g) and sodium chloride (0.38 g) were dissolved in 30 mL of DI water and stirred for 30 min. Subsequently, HCl (3 m) was added dropwise until the pH reached approximately 2, yielding a greenish‐yellow solution. The solution was transferred to a Teflon‐lined stainless‐steel autoclave and heated at 180°C for 24 h. After cooling to room temperature, the resulting WO_3_ precipitate was washed repeatedly with DI water and dried at 200 °C for 4 h.

### 3.3 Fabrication of MXene/ZnO NRs:WO_3_/CNT Cotton Fabric

Cotton fabric was initially cleaned by immersion in 0.1 m NaOH solution to remove surface impurities. After thorough rinsing with DI water, the fabric was dried and subsequently dip‐coated in MXene dispersion five times, with each side immersed for 2 min/cycle at room temperature, followed by drying at 60°C for 20 min.

To grow WO_3_‐doped ZnO NRs on the MXene‐coated fabric, Zn(NO_3_)_2_·6H_2_O (2.97 g) and hexamethylenetetramine (1.1 g) were dissolved in 25 mL of DI water. The solution was heated to 45°C, transferred to a Teflon‐lined autoclave, and supplemented with WO_3_ powder (5 mg). The MXene‐coated fabric was immersed in the precursor solution and maintained at 120°C for 6 h to facilitate the hydrothermal growth of ZnO NRs. Subsequently, the resulting MXene/ZnO NRs:WO_3_‐coated fabric was washed with DI water and dried at 100°C for 2 h. Finally, the fabric was immersed in a 0.1 wt% CNT solution for 15 min at room temperature, followed by drying at 90°C for 30 min in a vacuum oven to obtain a multifunctional fabric.

#### 3.3.1 Characterization Techniques

Surface morphology and microstructure of the coated fabric were examined using field‐emission‐scanning electron microscopy (FE‐SEM; SIGMA 300, Carl Zeiss, Oberkochen, Germany). Elemental distribution was analyzed by energy‐dispersive X‐ray spectroscopy (EDX; Sirion, FEI) and X‐ray photoelectron spectroscopy (XPS; K‐alpha+, ThermoFisher Scientific) equipped with an Al Kα radiation source. The crystalline structure of WO_3_ and the elemental composition of the coating on the fabric were analyzed using X‐ray diffraction (XRD; New D8 Advance, Bruker AXS, USA). EMI SE was measured using a vector network analyzer (VNA; Agilent/HP 8720C, 50 MHz–20 GHz) in the X‐band range (8.2–12.4 GHz) using the WR‐90 rectangular waveguide method. Surface electrical resistance of the fabric was measured using a digital multimeter (15B+, 600 V CAT III, Fluke, USA). Strain‐sensing performance of the fabricated multifunctional fabric was evaluated using a high‐precision electrometer (6517B, Keithley, USA) at room temperature. Thermal response was monitored via infrared thermal imaging (FLIR Pro, FLIR, USA). Thermogravimetric analysis (TGA) was performed using a NETZSCH TG 209F1 Libra analyzer from room temperature to 700°C at a heating rate of 10°C/min under a nitrogen atmosphere. Strain‐sensing performance of cotton fabrics were assessed using Rheometer (Sun Scientific Co.,Ltd, Japan).

## 4 Results and Discussion

### 4.1 Fabrication of Sandwich‐Structured Coatings Onto a Cotton Fabric

**FIGURE 1:**
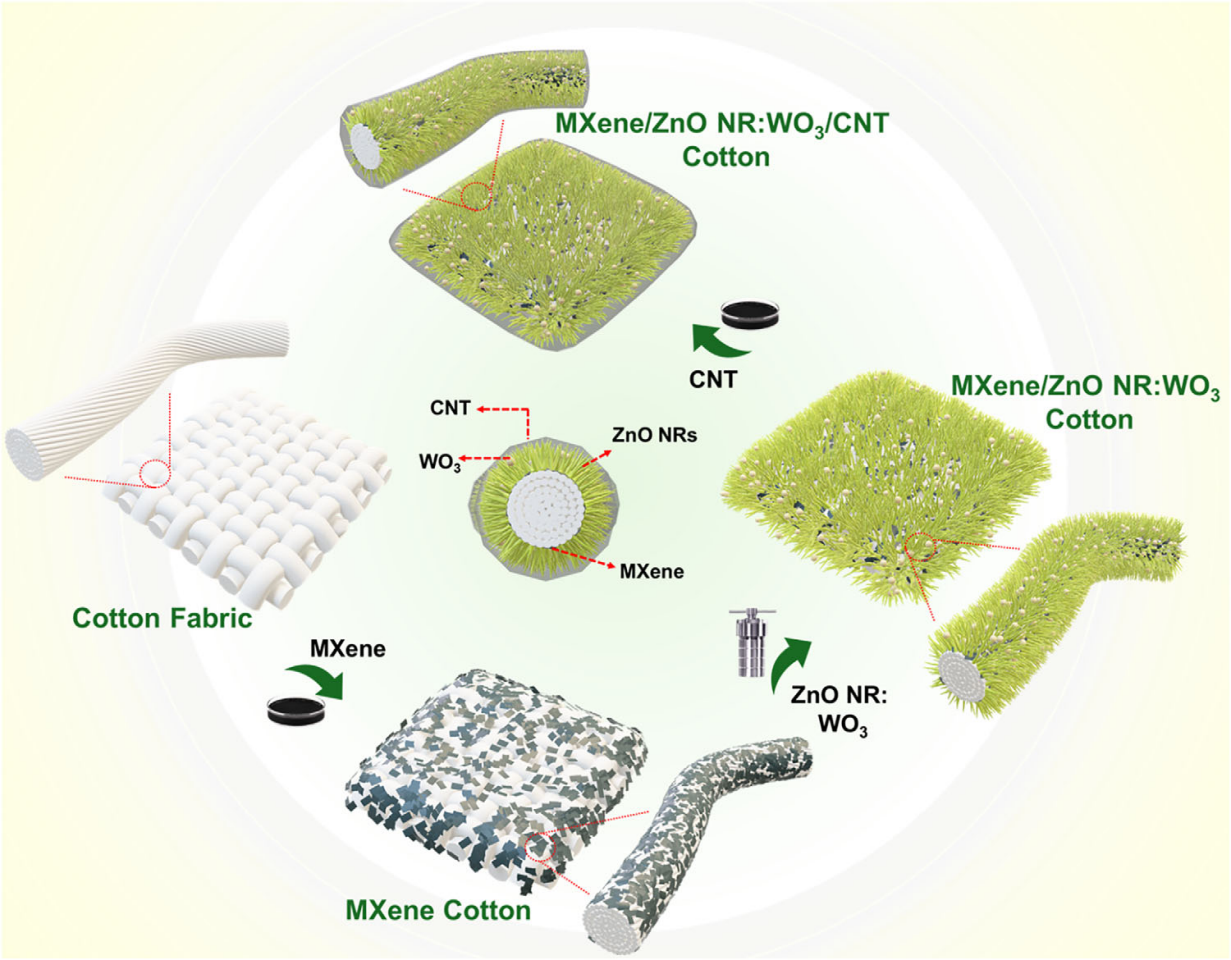
Schematic of the fabrication process for the trilayer sandwich‐structured MXene/ZnO NRs:WO_3_/CNT‐coated cotton fabric designed for multifunctional applications.

To endow cotton fabric with advanced multifunctionality, we engineered a sandwich‐structured coating composed of MXene, WO_3_‐doped ZnO NRs, and CNTs. This hierarchical architecture synergistically integrates the high electrical conductivity and EMI shielding capability of both MXene and CNTs, the semiconducting and piezoelectric properties of ZnO NRs: WO_3_ for improved carrier mobility and dielectric polarization, and the inherent mechanical flexibility of the textile substrate. The sandwich‐structured coating strategy is based on three design principles: (1) establishing a continuous MXene base layer to enable robust charge transport and electromagnetic attenuation [32]; (2) incorporating vertically aligned WO_3_‐doped ZnO NRs to introduce dielectric contrast, strain‐responsive piezoelectric behavior, and thermal sensitivity [33, 34, 35]; and (3) applying a CNT top layer to reinforce the conductive network, enhance mechanical durability, and provide additional EMI shielding via absorption and reflection pathways [36, 37]. This trilayer coating imparts the fabric with integrated functionalities including excellent Joule heating for wearable thermal therapy, sensitive strain sensing for real‐time human motion monitoring, and outstanding EMI shielding performance. Beyond these capabilities, the fabric was used as a smart safety interface capable of detecting unauthorized physical contact along designated boundaries and triggering immediate alerts. Additionally, the fabric was used to transmit Morse code signals in emergency communication scenarios. The stepwise fabrication of this trilayer‐coated cotton fabric is illustrated in Figure 1. The hierarchical architecture was constructed using sequential deposition techniques, each governed by tailored interfacial interactions. Initially, Ti_3_C_2_T_x_ MXene nanosheets, rich in surface terminations such as ─OH, ─F, and ─O, adhere to the hydroxyl‐rich cellulose fibers of cotton via hydrogen bonding and electrostatic interactions [38, 39], forming a uniform conductive base layer. Subsequently, WO_3_‐doped ZnO NRs are hydrothermally grown directly onto the MXene‐coated fabric. The MXene layer facilitates the nucleation and vertical alignment of ZnO NRs, and WO_3_ doping introduces lattice defects that enhance crystallinity and interfacial adhesion [40]. Finally, CNTs are deposited onto the ZnO NR:WO_3_@MXene‐coated fabric. Adhering via π–π stacking and van der Waals interactions to form a continuous, interconnected conductive network that conformally wraps around the underlying micro/nanostructures. This architecture markedly improves the electrical conductivity and imparts mechanical robustness to the coated textile [41], thereby enabling its multifunctional applications in wearable electronics, EMI shielding, Joule heating, and real‐time motion sensing. Fundamental Characterization of the as‐prepared MXene and WO_3_ is provided in (Figures S1 and S2).

### 4.2 Surface Characterization of Modified Cotton Fabrics

**FIGURE 2:**
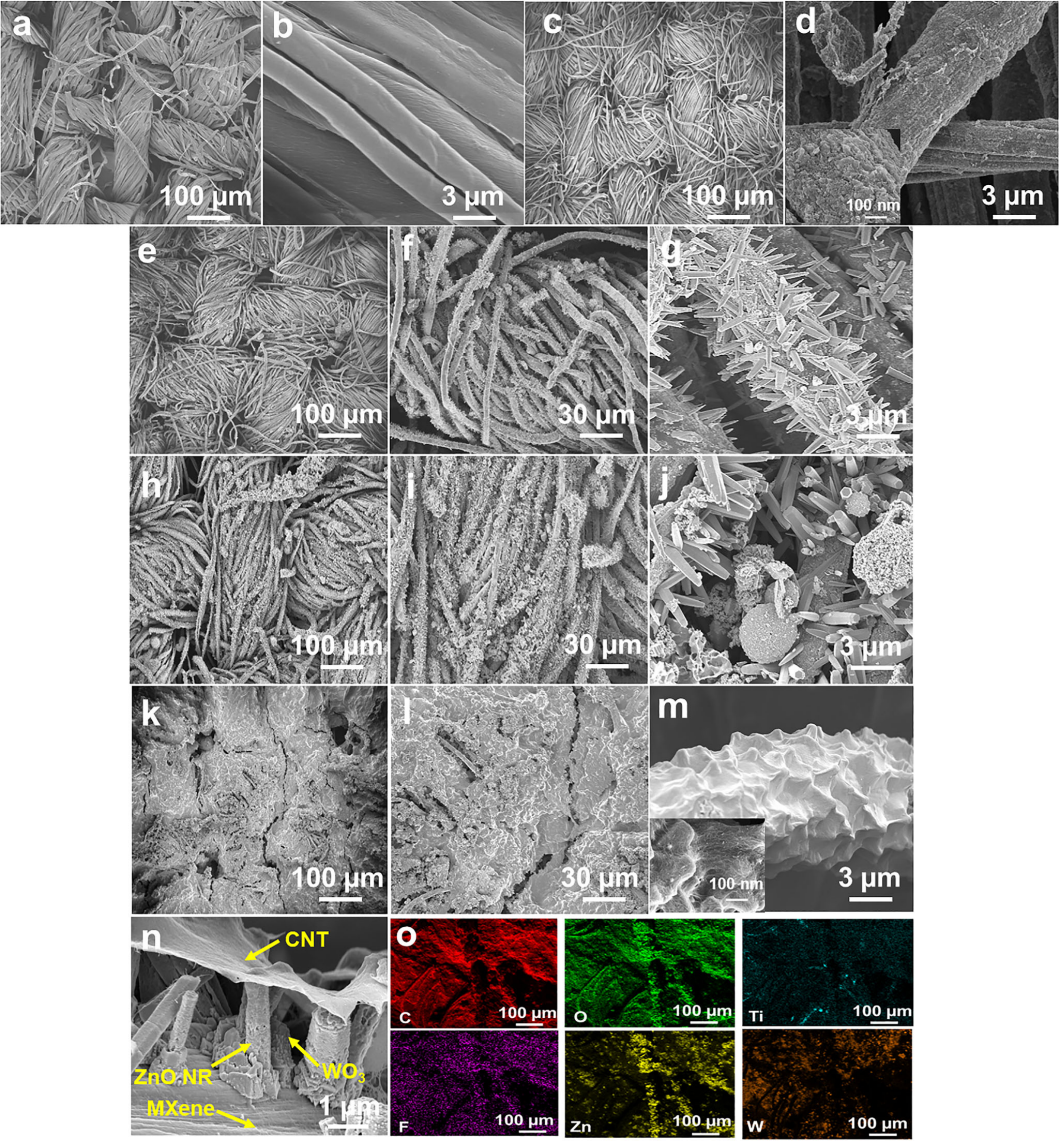
SEM images of (a, b) normal cotton; (c, d) MXene cotton; (e–g) Mxene‐ZnO NR cotton; (h–j) MXene‐ZnO NR:WO_3_ cotton; and (k‐m) MXene‐ZnO NR:WO_3_/CNT cotton. (n) Cross‐sectional SEM image of the trilayer coating on the fabric. (o) EDX elemental mapping of MXene‐ZnO NR:WO_3_/CNT‐coated fabric.

The distributions of MXene, CNTs, and hydrothermally grown ZnO NRs, along with the uniform incorporation of WO_3_, were critical determinants of the overall performance in multifunctional applications. Therefore, the morphological changes in the fabric after each sequential deposition step were systematically examined using FE‐SEM (Figure 2). The uncoated pristine cotton fabric exhibited a smooth, twisted fiber morphology with well‐defined inter‐fiber gaps (Figures 2a,b). After MXene deposition, a conformal and uniform coating layer was observed along the fiber surfaces (Figures 2c,d). A high‐magnification image (inset, Figure 2m) further revealed hierarchical surface morphology, with MXene sheets tightly wrapped around the fibers, confirming successful adsorption and intimate integration of the nanosheets. Subsequent hydrothermal growth of ZnO NRs resulted in a vertically aligned and uniform array across the MXene‐coated fiber surface, indicating directional growth and robust interfacial alignment of the NRs with the underlying conductive MXene layer (Figures 2e–g). Additionally, the presence of plate‐like WO_3_ structures distributed within and between the ZnO NRs in the MXene/ZnO NRs:WO_3_‐coated fabric validated the successful incorporation of WO_3_ into the ZnO matrix (Figures 2h–j). Finally, the deposition of CNTs over the MXene/ZnO NRs:WO_3_‐coated fabric resulted in a uniform, entangled CNT network (Figures 2k–m), conformally wrapping the ZnO NR architecture while preserving its hierarchical morphology. The high‐magnification SEM image (Figure 2m) revealed an intricate and densely entangled CNT network, uniformly distributed across the ZnO NRs:WO_3_‐modified textile surface.

To further validate the sandwich‐like configuration, cross‐sectional SEM imaging was conducted. The formation of a three‐layered structure, comprising MXene as the base layer, vertically aligned ZnO NRs as the intermediate scaffold, and CNTs as the outer conductive sheath, was validated. Each layer demonstrated a uniform, well‐integrated coating along the fiber surface (Figure 2n). Furthermore, to verify the homogeneous elemental distribution within the trilayer coating, EDX elemental mapping was performed. The EDX elemental maps of both uncoated and coated cotton fabrics are illustrated in Figures 2o and S3. The results confirm the successful deposition and uniform spatial distribution of each functional component, thereby validating the compositional uniformity and effective multilayer integration of the trilayer coating architecture on the cotton surface.

**FIGURE 3:**
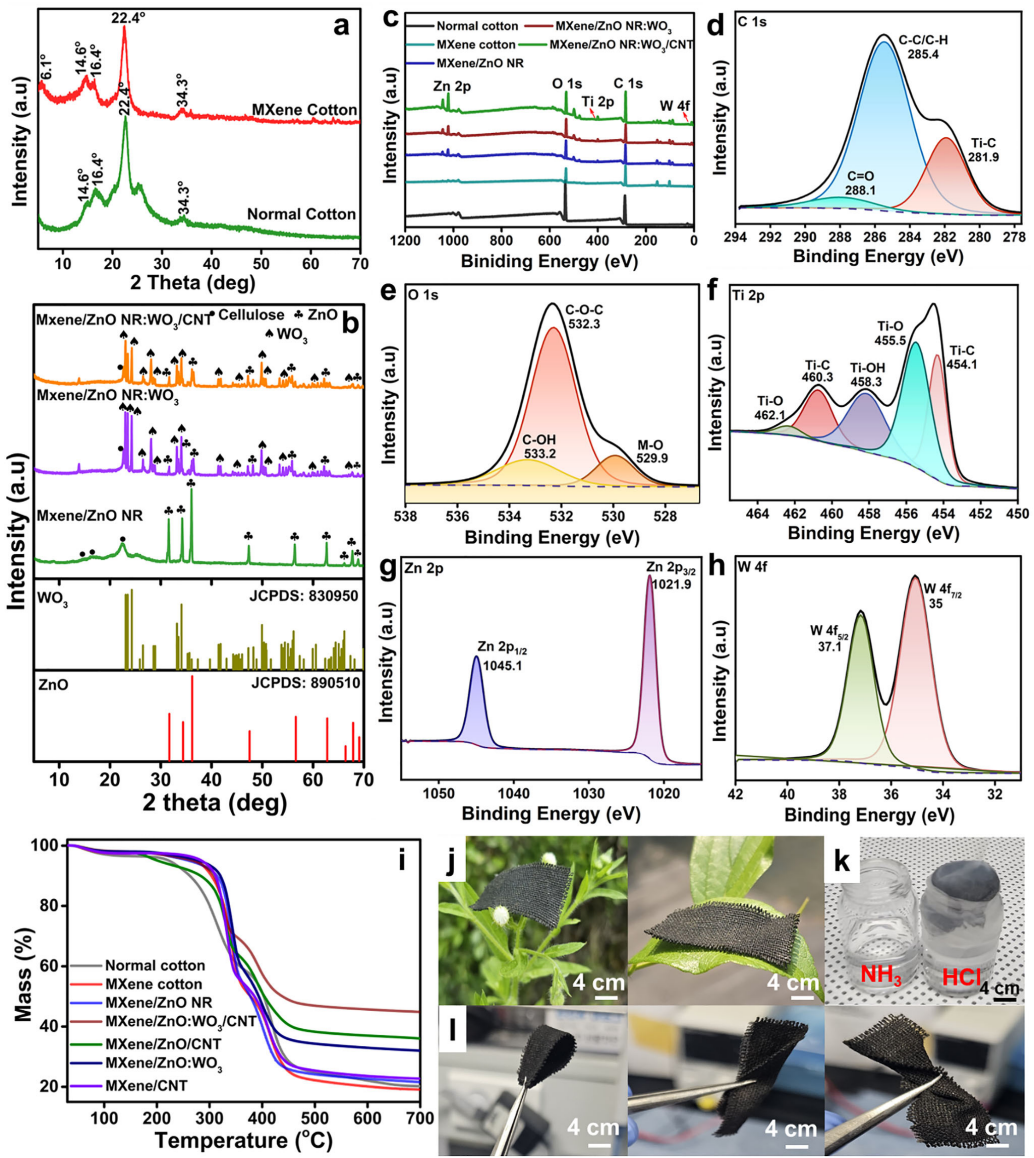
(a) XRD patterns of pristine and MXene‐coated cotton. (b) XRD patterns of MXene/ZnO NRs, MXene/ZnO NRs:WO_3_, and MXene/ZnO NRs:WO_3_/CNT‐coated cotton. (c) XPS survey spectra of coated and uncoated cotton samples. High‐resolution XPS spectra of (d) C 1s, (e) O 1s, (f) Ti 2p, (g) Zn 2p, and (h) W 4f for MXene/ZnO NRs:WO_3_/CNT‐coated cotton fabric. (i) TGA curves of surface‐modified cotton samples. Photographs demonstrating the (j) light weight, (k) breathable, and (l) flexible characteristics of MXene/ZnO NRs:WO_3_/CNT‐coated cotton.

The functionalization of cotton fabric with MXene, ZnO NRs, WO_3_, and CNTs was further validated via XRD and XPS analyses. As depicted in Figure 3a, pristine cotton exhibited characteristic XRD peaks at 2θ = 14.8°, 16.4°, 22.4°, and 34.7°, corresponding to the (110), (110), (002), and (004) crystal planes of cellulose I, respectively [42]. Notably, a peak around 2θ = 6.1° appeared in the MXene‐coated sample, corresponding to the (002) plane of Ti_3_C_2_T_x_ MXene [43], confirming the successful deposition of exfoliated MXene sheets onto the fabric surface. Importantly, the cellulose‐related peaks, along with the MXene peak, remained visible in both MXene and MXene/ZnO NR‐coated fabrics, indicating that the coatings preserved the crystalline structure of the cotton substrate. However, following the introduction of WO_3_‐doped ZnO NRs and the subsequent deposition of CNTs (Figure 3b), the diffraction peaks associated with cellulose and MXene became significantly diminished or entirely absent, suggesting the formation of a conformal, multilayered coating that obscured the native cotton structure. In these samples, additional intense peaks were observed at 2θ = 31.9°, 34.6°, 36.4°, 47.8°, 56.7°, 62.8°, 66.5°, 67.9°, and 69.3°, corresponding to the (100), (002), (101), (102), (110), (103), (200), (112), and (201) planes of the hexagonal wurtzite ZnO phase (JCPDS No. 89–0510) [44]. Distinct peaks corresponding to monoclinic WO_3_ (JCPDS No. 83–0950) were identified at 2θ = 23.1° (002), 23.4° (020), 24.5° (200), 25.6° (120), 27.9° (112), 33.2° (022), 33.9° (202), 35.7° (122), 42.1° (222), 47.5° (040), 49.9° (114), 55.8° (420), and 62.8° (340) [45], further confirming the successful incorporation of WO_3_ within the hybrid nanostructure. The combined presence of ZnO and WO_3_ phases provides strong evidence for the successful deposition of ZnO NR and WO_3_ onto the MXene‐coated cotton fabric.

The elemental composition and chemical bonding states of the trilayer coating on the cotton fabric were analyzed using XPS, as illustrated in Figure 3. The survey spectra (Figure 3c) revealed prominent peaks at approximately 284, 531, 1021, and 1045 eV, corresponding to the C 1s, O 1s, Zn 2p_3_/_2_, and Zn 2p_1_/_2_ orbitals, respectively. Additionally, low‐intensity peaks were observed at approximately 454 eV and between 34–38 eV, attributed to the Ti 2p and W 4f orbitals, respectively, confirming the successful deposition and integration of MXene, ZnO NRs, WO_3_, and CNTs onto the cotton fabric. The high‐resolution XPS spectra of each element in the MXene/ZnO NRs:WO_3_/CNT‐coated fabric were curve‐fitted, as shown in Figure 3d–h. The high‐resolution C 1s spectrum (Figure 3d) of the MXene/ZnO:WO_3_/CNT‐coated fabric displayed three distinct peaks, indicating the presence of multiple carbon environments. The dominant peak at 285.4 eV attributed to delocalized sp^2^‐hybridized carbon atoms, typically found in graphitic structures. Additional peaks at 288.1 and 281.9 eV corresponded to carbonyl (C═O) groups and C─Ti─O bonds, respectively, providing strong evidence for the successful integration of MXene onto the cotton surface. The O 1s spectrum (Figure 3e) exhibited three subpeaks at 529.9.1, 532.3, and 533.2 eV. The peak at 529.9 eV is attributed to the metal‐oxygen (M‐O) bonds (Ti─O, Zn─O and W─O bonds) from the MXene and ZnO NRs:WO_3_ framework, and those at 532.3 and 533.2 eV corresponded to oxygen‐containing groups such as C─O─C and C─OH (adsorbed hydroxyl/oxygenated surface groups), respectively.

In the Ti 2p region (Figure 3f), five subpeaks were identified. Peaks at 454.1 and 460.3 eV corresponded to Ti─C bonds, and those at 455.5 and 462.1 eV were attributed to Ti─O bonds. A distinct peak at 458.3 eV suggested the presence of surface hydroxyl groups (Ti─OH), indicating enhanced interfacial interaction with the cotton fiber due to surface functionalization. The Zn 2p spectrum (Figure 3g) exhibited a characteristic doublet with Zn 2p_3_/_2_ and Zn 2p_1_/_2_ peaks at 1021.9 and 1045.1 eV, respectively, demonstrating a spin–orbit splitting of 23.2 eV, consistent with Zn^2^^+^ in ZnO. Similarly, the W 4f spectrum exhibited two well‐defined peaks at 35 (W 4f_7_/_2_) and 37.1 (W 4f_5_/_2_) eV, with a spin–orbit splitting of 2.1 eV, confirming the incorporation of WO_3_ nanostructures within the ZnO NRs. These comprehensive XPS analyses further validate the successful formation of the MXene/ZnO:WO_3_/CNT sandwich structure on the cotton fabric surface. The combined presence of C─Ti (C 1s), Ti─O and Zn─O (O 1s and metal regions), and the O 1s component at ∼532.3 eV (C─O─C) indicates chemical coupling between cotton and MXene and interfacial interactions between MXene and ZnO:WO_3_. These spectral features, together with the metal oxide and carbon signatures, support formation of a stable MXene/ZnO:WO_3_/CNT sandwich structure on the cotton surface.

Thermal resistance and stability of the various functional coatings on cotton fabric were analyzed using TGA analysis (Figure 3i). The pristine cotton fabric exhibited primary degradation between 280°C and 350°C, corresponding to cellulose pyrolysis. A second major decomposition phase occurred at approximately 430°C, accompanied by significant weight loss. Conversely, the MXene‐coated cotton fabric displayed enhanced thermal resistance, evidenced by a slight shift of the initial degradation temperature to a higher range. This enhancement is attributed to the formation of a thermally stable inorganic MXene layer, which acts as a physical barrier that impedes heat and mass transfer during thermal decomposition. Further coatings with ZnO:WO_3_ and CNTs resulted in a progressive enhancement in thermal stability. ZnO NRs and WO_3_ nanoparticles functioned as ceramic fillers to promote char formation, and the outer CNT network formed a thermally conductive, entangled layer that promoted thermal shielding and suppressed volatile release. To specifically evaluate the role of ZnO NRs and WO_3_, a comparison was made with MXene/CNT‐coated cotton fabric (excluding ZnO NRs and WO_3_). The trilayer‐coated fabric (MXene/ZnO:WO_3_/CNT) exhibited the highest thermal stability among all the samples, with a significantly delayed onset of decomposition and a residual char yield of approximately 45% at 700°C, indicating substantial residue formation. These findings confirm the critical role of ZnO NRs and WO_3_ in reinforcing the thermal durability of the composite coating. The synergistic trilayer architecture integrates the structural robustness of ZnO NRs, the barrier efficiency of MXene, and the char‐forming, conductive nature of CNTs to substantially enhance the thermal stability of the cotton fabric.

To further demonstrate the mechanical flexibility, lightweight nature, and breathability of the multifunctional cotton fabric, a series of simple yet effective visual demonstrations were conducted. To highlight the lightweight character, the trilayer‐coated textile was delicately placed on soft substrates such as a flower petal and a leaf (Figure 3j). The ability of the fabric to rest without causing visible deformation or collapse of these fragile surfaces strongly confirms the ultralight properties of the fabric, an essential property for wearable electronic textiles. The breathability of the fabric was further demonstrated via a gas‐permeation test using HCl and ammonia (NH_3_). When an HCl bottle was covered with the MXene/ZnO:WO_3_/CNT‐coated fabric and exposed to NH_3_ vapor, characteristic white fumes of ammonium chloride (NH_4_Cl) were observed forming on the outer surface (Figure 3k). These findings indicate that gaseous molecules diffuse through the coated fabric, confirming their breathable properties despite the presence of functional multilayers. The flexibility of the fabric was verified by bending, twisting, and folding it into various configurations, as shown in Figure 3l. These results underscore the mechanical adaptability of the material for wearable and conformal applications. Such breathable yet functional textiles are promising candidates for prolonged skin contact and thermal comfort in wearable sensor applications.

### 4.3 Electrical Conductivity and EMI Shielding Performance of the Multifunctional Cotton Fabric

The rapid proliferation of portable and interconnected electronic devices has significantly increased electromagnetic (EM) radiation, raising serious concerns not only regarding device functionality but also human health. To address these challenges, the development of lightweight, flexible textiles with effective EMI shielding capabilities has become increasingly essential in modern daily life. According to Simon's theoretical formalism, EMI SE is closely related to both the electrical conductivity and thickness of the shielding material. Enhancing the electrical conductivity of cotton fabrics using functional coatings, therefore, offers a promising strategy for imparting EMI shielding functionality. For evaluation, the sheet resistance of the cotton fabric was measured after each stage of functional coating. Owing to the inherently insulating nature of pristine cotton, its sheet resistance could not be measured. After the initial MXene coating, a high sheet resistance of approximately 14 MΩ/sq was recorded. With each successive coating cycle, the conductivity progressively improved, reaching a substantially reduced sheet resistance of approximately 230 Ω/Sq after five coating cycles. Beyond this point, additional coating cycles did not result in significant improvement in sheet resistance, indicating a saturation threshold. Accordingly, five MXene coating cycles were optimized as the standard for subsequent experiments. Subsequent hydrothermal growth of ZnO NRs resulted in a moderate increase in sheet resistance to approximately 560 Ω/sq, which is attributed to the semiconducting nature of ZnO. Further incorporation of WO_3_ resulted in a slight increase in sheet resistance to approximately 600 Ω/sq, which can be attributed to its comparatively lower electrical conductivity and the introduction of additional interfacial resistance. The ZnO NR–WO_3_ growth parameters were optimized to ensure uniform surface coverage while maintaining electrical continuity, as evidenced by consistent sheet resistance values across the coated fabric. Subsequently, conductive CNTs were deposited as the outermost layer to restore and enhance the overall electrical conductivity. The CNT coating was applied through successive deposition cycles, and two cycles were identified as optimal, beyond which the sheet resistance reached a saturation state and no further significant reduction was observed. As a result, the final MXene/ZnO:WO_3_/CNT‐coated fabric exhibited a markedly reduced sheet resistance of approximately 15 Ω/sq, indicating the formation of an efficient and stable conductive network.

The EMI shielding performance of the prepared cotton fabric was systematically evaluated in the X‐band frequency range (8.2–12.4 GHz). For EMI shielding evaluation, all coated and uncoated cotton fabrics with dimensions of 2×2 cm^2^ (450 g m^−2^). The samples were mounted in the standard waveguide holder according to the measurement protocol, ensuring full coverage of the waveguide aperture. The total EMI SE can be evaluated using the scattering parameters and their associated reflection and transmission coefficients, as calculated by the following equations:

(1) $$R={\left|S_{11}\right|}^{2}$$

(2) $$T={\left|S_{21}\right|}^{2}$$

(3) A+R+T=1

(4) $$SE_{R}=-10\log\left(1-R\right)=-10\log\left(1-{\left|S_{11}\right|}^{2}\right)$$

(5) $$SE_{A}=-10\log\left(T/1-R\right)=-10\log\left({\left|S_{21}\right|}^{2}/1-{\left|S_{11}\right|}^{2}\right)$$

(6) $$SE_{T}=SE_{A}+SE_{R}+SE_{M}$$

$$SE_{T}>10\;\mathrm{dB},\;SE_{M}\;\mathrm{is}\;0$$

**FIGURE 4:**
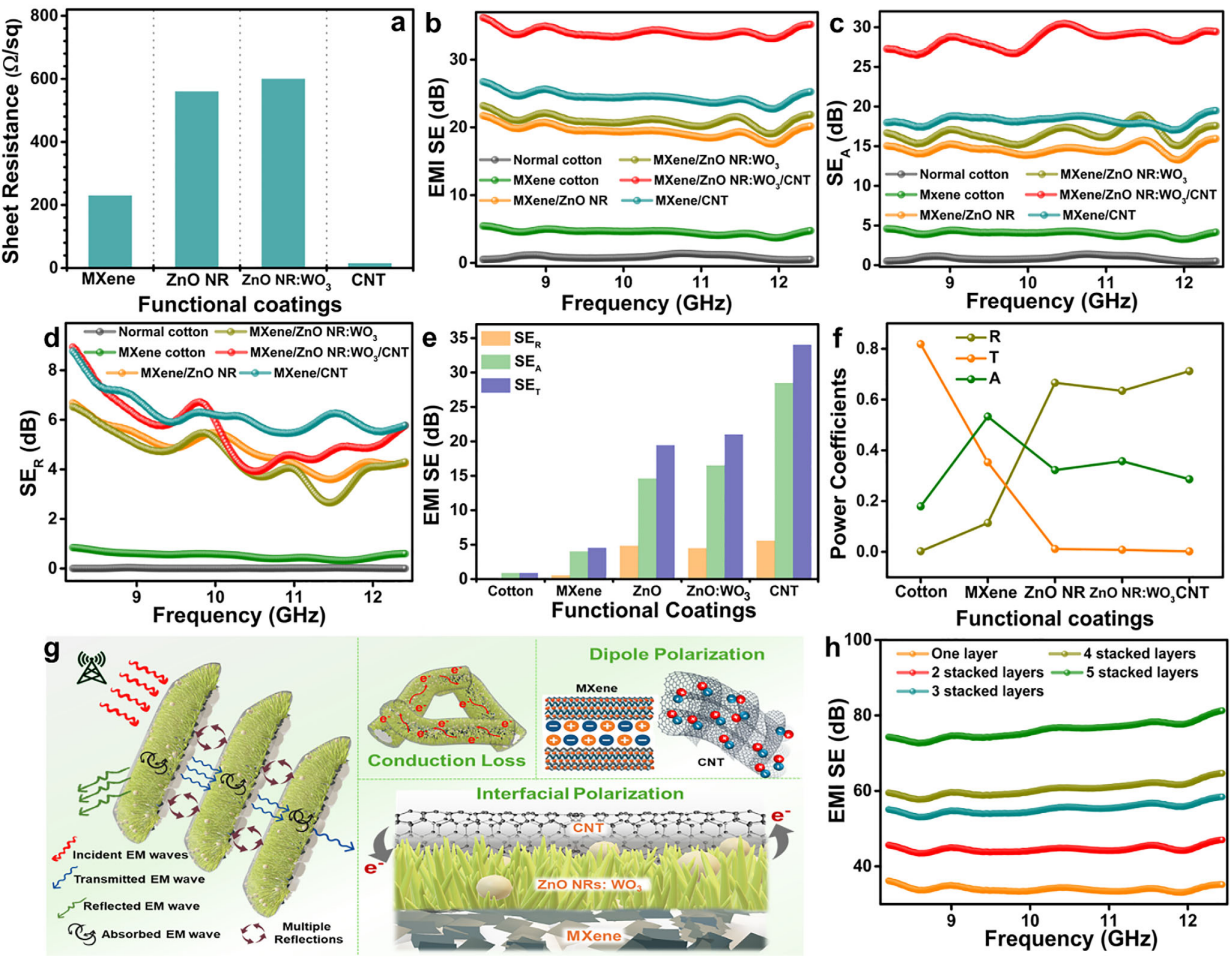
(a) Electrical resistance measurements of the coated cotton fabric. (b) Total EMI SE. (c) SE_A_. (d) SE_R_. (e) Average values of SE_T_, SE_A_, and SE_R_. (f) Average power coefficients of pristine and coated cotton fabric. (g) EMI shielding mechanism of MXene/ZnO NRs:WO_3_/CNT‐coated cotton fabric. (h) EMI SE of MXene/ZnO NRs:WO_3_/CNT‐coated cotton with “n” layers.

Owing to its electrically insulating properties, the pristine cotton with 0.12 mm exhibited significantly low EMI SE, with an average value of only 0.8 dB, indicating that electromagnetic waves can easily penetrate the fabric without significant attenuation. Although the functional coatings of MXene, ZnO NRs, and WO_3_‐doped ZnO NRs imparted moderate electrical conductivity to the cotton fabric, they significantly enhanced its EMI SE. The MXene‐coated cotton, with a thickness of 0.17 mm, achieved a modest EMI SE of 4.5 dB, and the subsequent addition of ZnO NRs and ZnO NRs:WO_3_ further increased the coating thickness to 0.19 mm, resulting in EMI SE values of 19.4 and 20.9 dB, respectively. Notably, the ZnO NRs:WO_3_ coating enabled the fabric to surpass the practical EMI SE threshold of 20 dB, demonstrating its suitability for functional shielding applications. Significantly, with the final deposition of a highly conductive CNT layer, the overall thickness increased slightly to 0.21 mm; however, the trilayer MXene/ZnO:WO_3_/CNT‐coated cotton fabric exhibited a remarkable EMI SE of 34.4 dB (Figure 4b), corresponding to approximately 99.9% SE. The gradual increase in coating thickness also contributes to the enhanced EMI attenuation by extending the EMW propagation path and promoting multiple internal reflections within the coated fabric. This superior performance not only exceeds the benchmark for commercial EMI shielding textiles but also underscores the synergistic interplay between conductive and semiconductive layers in enhancing overall SE.

To further elucidate the role of semiconductive ZnO NRs and WO_3_, cotton fabric coated only with MXene and CNTs was analyzed. Although the fabric exhibited an EMI SE of 27.4 dB, the SE remained substantially lower than that of the trilayer MXene/ZnO NR:WO_3_/CNT‐coated fabric. This comparison underscores the synergistic contribution of ZnO NRs and WO_3_ in enhancing the EMI shielding performance of conductive textiles. These findings confirm the critical role of rationally engineered multilayer architectures in the development of high‐performance EMI shielding fabrics for wearable applications. The average EMI SE (SE_T_) of multifunctional cotton fabrics comprises three components: absorption effectiveness (SE_A_), reflection effectiveness (SE_R_), and multiple reflection effectiveness (SE_M_). However, the contribution of SE_M_ is typically negligible when SE_T_ exceeds 10 dB. Therefore, only SE_A_ and SE_R_ values were calculated for the coated cotton fabric, as presented in Figure 4c,d. Additionally, the average SE_T_, SE_A_, and SE_R_ values of the coated cotton fabric are summarized in Figure 4e. The results indicate that the SE_A_ value consistently exceeds the SE_R_ value across all functionalized samples, suggesting that electromagnetic wave attenuation primarily occurs via internal absorption rather than surface reflection. While the MXene/ZnO NRs: WO_3_/CNT‐coated cotton fabric exhibited a superior SE_A_ value, further analysis was conducted to clarify the electromagnetic attenuation mechanism associated with the coated materials. Accordingly, power coefficients such as absorption (A), reflection (R), and transmission (T) were computed to identify the dominant EMI shielding pathways contributing to the performance of the coated cotton fabric. The A, R, T values of the cotton sample are presented in Figure 4f. The results confirm that the applied sandwich‐structured coating onto the cotton fabric exhibits substantially low T = approximately 0.001 and high R = approximately 0.71. The increase in reflection after incorporation of the ZnO NRs:WO_3_ interlayer originates from the impedance mismatch introduced by the semiconducting ZnO NRs: WO_3_ between the highly conductive MXene and CNT networks, which promotes stronger interfacial reflection of incident electromagnetic waves. The EMI shielding behavior of the MXene/ZnO:WO_3_/CNT‐coated cotton fabric was therefore primarily governed by a reflection‐dominated mechanism. Thus, the multilayered coating synergistically integrates the electrical, dielectric, and interfacial properties of various materials to effectively suppress EM waves.

Owing to its excellent electrical conductivity and resulting impedance mismatch, the MXene coating formed a continuous, highly conductive network on the fabric surface, effectively reflecting most of the incoming EM radiation. Beyond reflection, the surface functional groups on MXene (─OH, ─F, and ─O) introduced dipolar polarization losses, enabling partial absorption of EM waves [46, 47]. The outermost layer of CNT coating enhanced SE by reinstating high surface conductivity. Under EM exposure, the delocalized charge carriers in CNTs experienced displacement, generating conduction losses. Simultaneously, structural defects and residual functional groups enhanced dipolar polarization [48]. The intermediate layer of WO_3_‐doped ZnO NRs introduced a semiconducting interface with pronounced dielectric contrast relative to the adjacent conductive layers, thereby promoting interfacial polarization. This effect is further supported by impedance spectroscopy results (Figure S4), where the ZnO: WO_3_‐containing textile exhibits a pronounced capacitive response and frequency‐dependent phase shift, indicating enhanced charge accumulation and polarization at heterogeneous interfaces. Such interface‐dominated electrical behavior facilitates increased EM energy dissipation through absorption mechanisms [49]. These combined effects enabled efficient attenuation of EM radiation via a balance of reflection and absorption. The hierarchical structure and conductivity gradient across the trilayer coating promoted successive reflection and scattering of EM waves at multiple internal interfaces. Thus, architecture reinforces the reflection‐dominant shielding pathway while simultaneously benefiting from conduction loss with supporting dielectric and polarization loss mechanisms (Figure 4g). Consequently, the MXene/ZnO:WO_3_/CNT‐coated cotton fabric demonstrates exceptional EMI shielding performance, making it a promising and multifunctional candidate for applications in next‐generation wearable and flexible electronics.

Further, the thickness of the coating influences its EMI SE value. For evaluation, the EMI SE of the coated cotton fabric was measured by stacking 1–5 fabric layers, as shown in Figure 4h. With each additional layer, a notable increase in the total EMI SE was observed, reaching approximately 78 dB for five layers. This enhancement demonstrates the effectiveness of the MXene/ZnO:WO_3_/CNT coating thickness for high‐performance EMI shielding applications. Furthermore, the durability of the MXene/ZnO:WO_3_/CNT coatings was systematically assessed under repeated bending cycles (Figure S5), demonstrating that the fabricated multifunctional cotton fabric retained stable EMI shielding performance, without significant degradation. To evaluate the oxidation stability of MXene, the EMI SE of the MXene/ZnO:WO_3_/CNT‐coated fabric was measured after storing it under ambient open‐air conditions for 30 d. The coated fabric maintained a consistently high EMI SE, suggesting that the trilayer architecture effectively suppressed surface oxidation of MXene, thereby preserving its shielding performance.

In addition, a comparative evaluation of EMI SE with previously reported EMI shielding textiles is provided in the Supporting Information (Table S1). Compared with conducting polymer‐based and other MXene‐modified fabrics, our design demonstrates excellent EMI shielding performance. While some MXene‐based textile platforms achieve higher EMI SE values (∼80 dB), they typically require dense multilayer coatings through repeated dipping processes, which compromise flexibility and breathability. In contrast, the proposed trilayer architecture delivers a balanced combination of conductivity, dielectric polarization, and structural compliance, enabling stable EMI shielding without sacrificing breathability or multifunctionality.

### 4.4 Joule Heating and De‐Icing Performance of the MXene/ZnO:WO_3_/CNT‐Coated Cotton Fabric

Wearable electronic fabrics with efficient electrical conductivity for thermal management in diverse climatic conditions are gaining increasing interest owing to their skin‐conformability, flexibility, and breathability. The proposed, highly conductive MXene/ZnO:WO_3_/CNT‐coated cotton fabric, featuring interconnected conductive networks, enables efficient Joule heating under applied voltage. This architecture facilitates rapid heat generation and uniform distribution, resulting in high electrothermal efficiency suitable for practical wearable heating applications.

**FIGURE 5:**
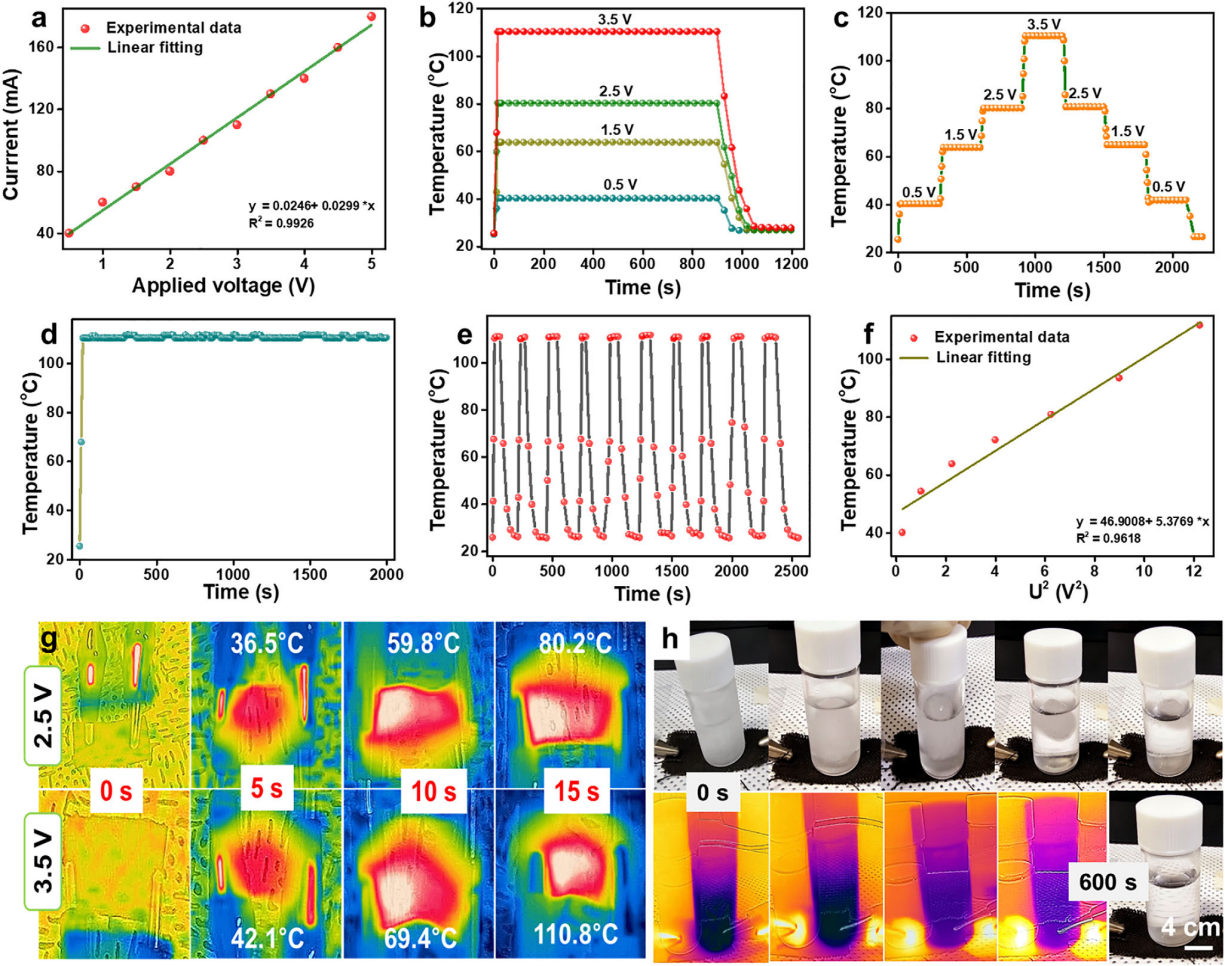
(a) I–V characteristics of MXene/ZnO NRs:WO_3_/CNT‐coated cotton fabric. (b) Temperature profile of the multifunctional cotton fabric. (c) Temperature response of MXene/ZnO NRs:WO_3_/CNT‐coated cotton fabric under stepwise increasing applied voltages. (d) Temperature stability of MXene/ZnO NRs:WO_3_/CNT‐coated cotton fabric at a constant voltage of 3.5 V. (e) Cyclic thermal stability at 3.5 V. (f) Relationship between equilibrium temperature and voltage (U^2^) for the MXene/ZnO NRs:WO_3_/CNT fabric. (g) IR thermal images at different time intervals. h Photographs and IR thermal images showing the ice‐melting performance of the MXene/ZnO NRs:WO_3_/CNT‐coated cotton fabric.

For Joule heating studies, MXene/ZnO:WO_3_/CNT‐coated cotton fabric with dimensions of 2 × 2 cm^2^ (450 g m^−2^) and a thickness of 0.21 mm were used. The device was fabricated by directly applying electrical contacts to opposite ends of the coated fabric using conductive clips. A DC voltage was applied across the two ends without any additional encapsulation, and the Joule heating performance of the MXene/ZnO:WO_3_/CNT‐coated cotton fabric was investigated under ambient conditions. Electrothermal performance is governed by the Joule heating effect, wherein heat is directly generated by the flow of electric current through conductive textiles, ensuring rapid thermal response. The I–V characteristics of the MXene/ZnO:WO_3_/CNT‐coated cotton fabric were evaluated to assess its electrical behavior under applied voltage, as shown in Figure 5a. The results demonstrate a linear increase in current with voltage, indicating stable ohmic contact and reliable conductive pathways across the multilayer structure. Changes in surface temperature as a function of time under applied voltages ranging from 0.5 to 3.5 V are presented in Figure 5b. The fabric demonstrated rapid heating, attaining a steady‐state temperature within ∼15 s at all testing voltages (Figure S7). Therefore, owing to the high electrical conductivity of the MXene/ZnO:WO_3_/CNT‐coated cotton fabric, excellent Joule heating performance under low applied voltage was observed.

For comparison, the Joule heating performance of MXene/CNT‐coated cotton fabric was evaluated under identical conditions to clarify the contribution of the ZnO:WO_3_ interlayer. Despite exhibiting a low sheet resistance (∼23 Ω), the MXene/CNT fabric required a longer time (∼40 s) to reach a surface temperature of ∼ 90.7°C at an applied voltage of 3.5 V (Figure S6). In contrast, the MXene/ZnO:WO_3_/CNT‐coated fabric achieved higher temperatures within a shorter time frame under the same operating conditions. This enhancement indicates that the presence of the ZnO:WO_3_ interlayer modulates charge transport and energy dissipation behavior through the formation of heterogeneous interfaces [50].

The stepwise electrothermal stability of the MXene/ZnO:WO_3_/CNT‐coated cotton fabric is shown in Figure 5c. The applied voltage gradually increased from 0.5 to 3.5 V and then reduced to 0.5 V. Accordingly, the surface temperature rose incrementally, reaching a peak at approximately 110°C for 3.5 V. This linear and reversible temperature progression with voltage underscores the controllable and tunable Joule heating performance of the fabric. The consistent heating and cooling behavior upon voltage cycling demonstrates excellent electrothermal reversibility and operational stability of the multifunctional cotton fabric. It should be noted that the cooling behavior observed in Figure 5c differs from that in Figure 5b due to the distinct voltage control modes employed. In Figure 5c, the applied voltage is reduced stepwise rather than being completely switched off, resulting in residual Joule heating at each voltage step. This partial heat generation counteracts heat dissipation to the surroundings, leading to a gradual and smooth cooling profile. In contrast, Figure 5b represents abrupt voltage shut off (V → 0), where Joule heating is instantaneously terminated and the temperature decays rapidly, governed solely by natural heat dissipation. Furthermore, the long‐term Joule heating performance of the MXene/ZnO:WO_3_/CNT‐coated cotton fabric was evaluated to verify its durability under continuous operation. Upon applying a constant voltage of 3.5 V, the surface temperature remained stable at approximately 110.5°C for over 2000 s, without any significant thermal decay, and the corresponding infrared images captured at different intervals confirm uniform heat distribution across the fabric (Figure 5d).

Additionally, multiple Joule heating and cooling cycles were performed at a constant voltage of 3.5 V, as illustrated in Figure 5e. The fabric consistently attained stable temperatures across all 10 cycles, demonstrating excellent repeatability and electrothermal reliability essential for long‐term wearable applications. The relationship between the square of the applied voltage (U^2^) and the steady‐state surface temperature of the coated cotton fabric is shown in Figure 5f. Temperature exhibited a nearly linear increase with U^2^, demonstrating a controllable and predictable Joule heating behavior. The results confirm that the thermal output of the fabric can be precisely tuned by varying the input voltage. Furthermore, infrared (IR) thermal images of the multifunctional fabric captured at different intervals are shown in Figure 5g, validating the efficient and uniform Joule heating behavior across the fabric. A consistent thermal distribution underscores the strong electrical connectivity and excellent thermal responsiveness of the trilayer‐coated cotton fabric. The mechanical durability of the multifunctional coating was evaluated by subjecting the cotton fabric to 500 repeated bending cycles. After 500 bending cycles, the MXene/ZnO NRs:WO_3_/CNT‐coated cotton fabric retained comparable Joule heating performance and exhibited stable cyclic heating‐cooling behavior, demonstrating good mechanical durability and electrical stability of the multifunctional coating (Figure S8).

Given the excellent and stable Joule heating performance, the de‐icing capability of the MXene/ZnO NR:WO_3_/CNT‐coated cotton fabric was evaluated to demonstrate its practical applicability in extreme environments. To assess the de‐icing performance of MXene/ZnO NR:WO_3_/CNT‐coated fabric (Figure 5h), an ice‐filled bottle was placed in direct contact with the fabric, and a constant voltage of 4 V was applied. For de‐icing demonstrations, MXene/ZnO NR:WO_3_/CNT‐coated fabric with dimensions of 4 × 2.5 cm (200 g m^−2^) was employed. Voltage was applied directly across the two ends of the fabric, similar to the Joule heating configuration, to induce uniform electrothermal heating. Owing to the rapid electrothermal response of the fabric, visible melting began within 25 s, and the ice was entirely liquefied within 10 min. Conversely, under ambient conditions without heating, the ice melted within approximately 25–30 min. This significant reduction in melting time highlights the potential of the fabric for active de‐icing applications in wearable electronics.

A comprehensive comparison of the Joule heating performance of the MXene/ZnO NRs:WO_3_/CNT‐coated cotton fabric with previously reported conductive textiles is presented in Table S2. In contrast to earlier studies, the proposed trilayer fabric attains 110°C within a notably short response time of ∼15 s, thereby demonstrating superior electrothermal performance characterized by rapid heating, low‐voltage operation, and enhanced flexibility. These attributes underscore its potential for advanced wearable thermal management and safety applications.

### 4.5 Strain Sensing and Real‐time human Motion Monitoring Performance of MXene/ZnO NR:WO_3_/CNT Cotton Fabric

Owing to their excellent flexibility, light weight, and ability to conform to the human body, textile‐based strain sensors are ideal for integration into wearable devices. Their breathability and comfort make them particularly suitable for critical applications such as real‐time health monitoring, rehabilitation tracking, and sports performance analysis. Existing studies have explored the individual contributions of MXene, CNTs, and ZnO nanostructures in enhancing strain‐sensing performance, based on their distinct electrical, mechanical, and piezoresistive properties. Inspired by those performances, the present work integrates all three components into a trilayer coating on a cotton fabric, resulting in superior strain sensitivity, improved structural integrity, and enhanced responsiveness to various mechanical deformations.

**FIGURE 6:**
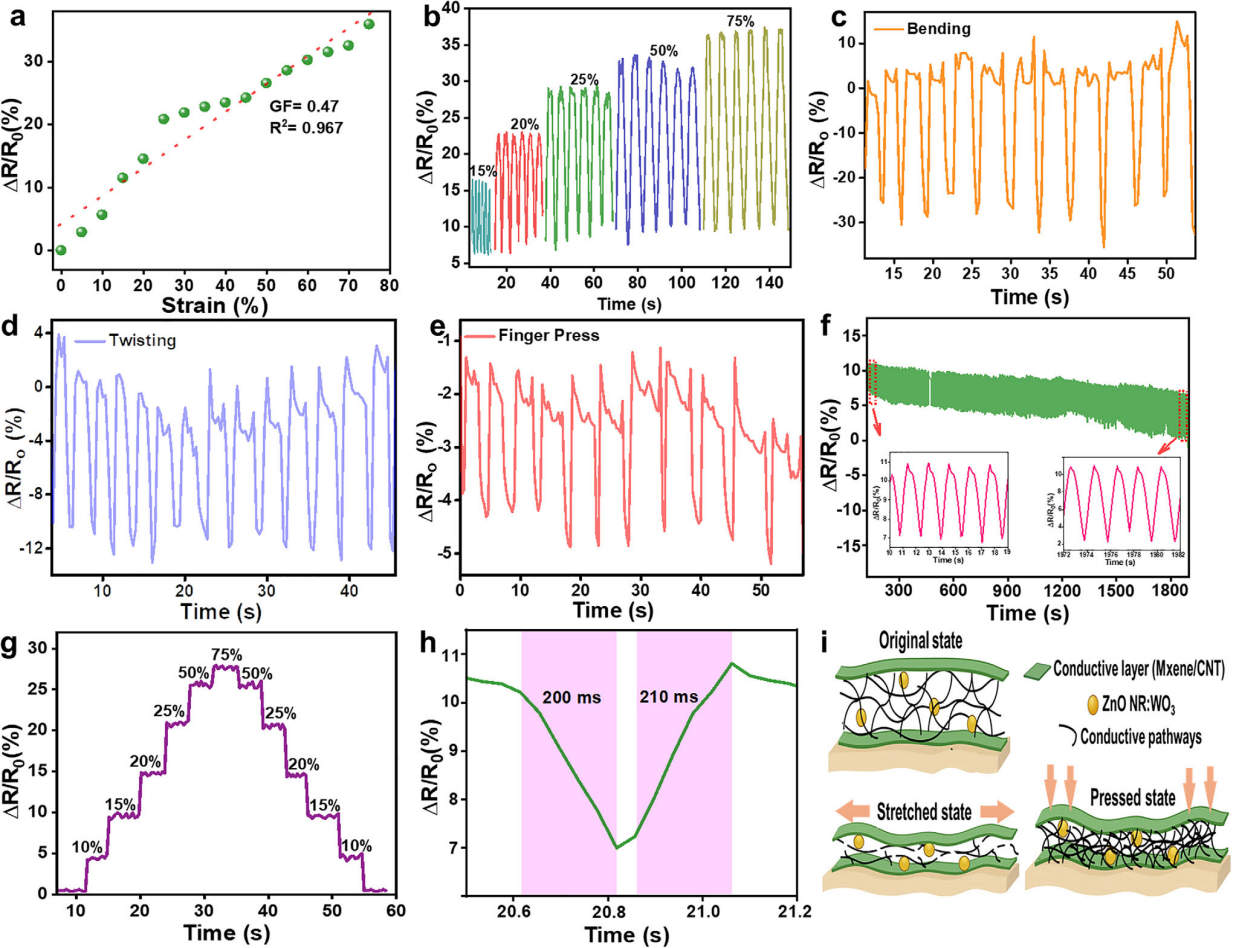
Sensing performance of MXene/ZnO NR:WO_3_/CNT‐coated cotton fabric. (a) Relative resistance change under different strain levels. (b) Relative resistance change under various stretching states. Resistance response under different (c) bending, (d) twisting, and (e) finger pressing cycles. (f) Strain‐sensing stability under 2000 cycles of stretching and releasing at 10% strain. (g) Relative resistance change under stretching. (h) Response and recovery time as a strain sensor. (i) Schematic of the conductive network changes during stretching and pressing.

To evaluate the strain‐sensing capability of the sandwich‐structured MXene/ZnO NR: WO_3_/CNT‐coated cotton fabric with dimensions of 3 × 2 cm^2^ (300 g m^−2^) were used, the performance of the sensor was characterized based on its sensitivity and calculated gauge factor (GF) under various applied tensile strains. The sensing behavior was assessed by monitoring the relative change in electrical resistance, defined as (R − R_0_)/R_0_, where R_0_ is the initial resistance of the fabric in its unstrained state and R is the resistance under applied strain. The GF, which quantifies the sensitivity of the sensor to mechanical deformation, was determined by correlating the relative resistance change to the applied strain using following relationship: GF = (R − R_0_)/R_0_*𝜀. Here, 𝜀 represents the applied tensile strain. Figure 6a illustrates the relative resistance variation of the MXene/ZnO NR:WO_3_/CNT‐coated cotton fabric under tensile strain ranging from 0% to 75%. The multifunctional fabric exhibits a GF of 0.47, which is notably higher than that of the MXene/CNT‐coated cotton fabric (GF = 0.25, Figure S9), indicating enhanced strain sensitivity. This improvement reflects the more pronounced electromechanical response of the MXene/ZnO NR:WO_3_/CNT trilayer architecture compared to the MXene/CNT system across a wide range of mechanical deformations. Furthermore, the strain response of the MXene/ZnO NR:WO_3_/CNT‐coated cotton fabric was evaluated under various strains of 15%, 20%, 25%, 50%, and 75%. The corresponding relative resistance changes (ΔR/R_0_ (%)) were recorded, as illustrated in Figure 6b. The results show that the relative resistance consistently increases with stretching rate, demonstrating the robustness and dynamic responsiveness of the sensor across different deformation speeds. The resistance variations were also studied under bending and twisting deformations, which were manually applied by hand, to evaluate the mechanical adaptability and durability of the sensor. For both bending and twisting cycles (Figures 6c,d), the MXene/ZnO NR:WO_3_/CNT‐coated cotton fabric exhibited consistent resistance responses, indicating excellent mechanical robustness and reliable sensing capability. Moreover, a noticeable and repeatable change in resistance was observed when the fabric was gently pressed using a finger (Figure 6e), further confirming its high sensitivity and responsiveness to mechanical stimuli. This stable behavior is attributed to the well‐integrated multilayer conductive network, which maintains its structural integrity under repeated mechanical deformation.

To further assess the electrical stability under prolonged mechanical stress, a cyclic bending test was conducted for over 2000 cycles at strain 10%. As shown in Figure 6f, the sensor maintained stable electrical resistance throughout the test without any noticeable drift or degradation, demonstrating its outstanding mechanical endurance and potential for long‐term wearable sensing applications. The response behavior was further assessed to evaluate the dynamic sensing capability of the fabric. Upon the application and release of strain, the resistance sharply increased to its peak value and promptly returned to the baseline (Figure 6g). Notably, the response and recovery times were 200 and 210 ms, respectively (Figure 6h), highlighting the ultrafast responsiveness and excellent reversibility of the sensor, which are essential for real‐time motion detection in wearable electronics. This excellent combination of fast response, stable recovery, and long‐term mechanical durability stems from the well‐engineered structure of the coated fabric.

Further, as detailed in the (Table S3), the proposed trilayer‐coated fabric demonstrates superior strain‐sensing performance compared to conventional textile‐based sensors, offering a well‐balanced combination of sensitivity, flexibility, and stability. The strain‐sensing capability originates from the synergistic interplay of conductive and semiconductive materials distributed across the flexible textile substrate. Under mechanical deformation, such as bending or stretching, the interconnected conductive pathways formed by MXene and CNTs undergo slight disruptions or reconnections (Figure 6i), leading to measurable variations in electrical resistance [51, 52, 53, 54]. Semiconductors such as ZnO exhibit inherently high strain sensitivity due to pronounced piezoresistive effects. Under mechanical deformation, the crystal lattice of a semiconductor experiences changes in interatomic spacing and band structure, leading to substantial modulation of charge carrier distribution and electrical resistivity. This effect is much stronger in semiconductors than in metals, because the change in resistivity under strain contributes significantly to the overall electrical response beyond mere geometric effects. In the present MXene/ZnO:WO_3_/CNT composite, the semiconducting ZnO:WO_3_ interlayer provides additional strain‐dependent resistance modulation, which when coupled with the percolated conductive network of MXene and CNTs amplifies the overall gauge factor and sensing response compared to a purely conductive network [55, 56, 57]. This multiscale strain‐responsive behavior enables the fabric to accurately detect low‐ and high‐strain movements. When placed on the human body, the fabric can reliably monitor various human motions such as finger bending, joint flexing, or subtle muscle contractions demonstrating its potential as a wearable strain sensor for real‐time motion monitoring.

**FIGURE 7:**
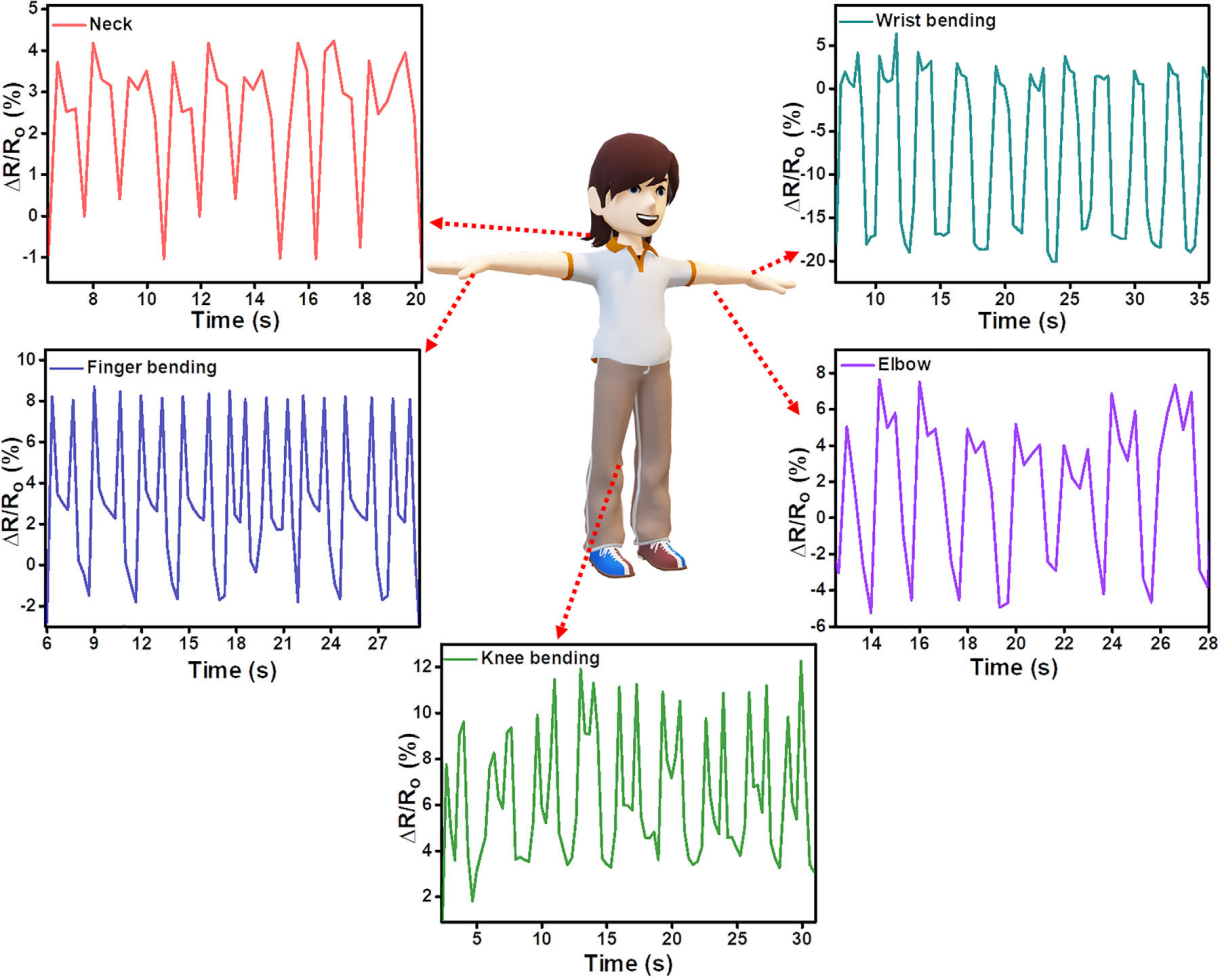
Real‐time human motion monitoring of MXene/ZnO NRs:WO_3_/CNT cotton fabric.

To demonstrate the practical applicability of the MXene/ZnO NR:WO_3_/CNT‐coated cotton fabric as a wearable strain sensor, its ability to detect a range of human body movements was systematically evaluated (Figure 7; video S1). The fabric sensor was directly attached to different parts of the human body, specifically the finger, wrist, elbow, knee, and neck, and subjected to controlled cyclic bending to assess signal reproducibility, strain sensitivity, and mechanical durability under real‐time physiological movements. During finger bending, the sensor showed clear and repeatable resistance responses, indicating its suitability for monitoring human‐motion‐induced deformation. For wrist movements, the sensor was attached across the joint to monitor pure‐bending motions. The resulting resistance signals were distinct and highly reproducible, demonstrating the ability of the sensor to detect moderate strain amplitudes generated by wrist flexion and extension. No degradation in signal quality was observed over successive cycles, underscoring its robustness under repetitive strain. Larger joint articulations, such as elbow and knee bending, imposed higher mechanical deformation. In these configurations, the sensor captured pronounced resistance variations with excellent linearity and stability across full‐range motion (e.g., up to 120° bending). These results affirm the dynamic response and mechanical compliance of the sensor under wide strain windows. To assess the response to subtle biomechanical activity, neck motions involving tilting and nodding were recorded. The sensor produced consistent resistance profiles even under low‐magnitude strain highlighting its sensitivity to delicate, low‐frequency movements such as those linked to posture or respiratory activity. Overall, the MXene/ZnO NR:WO_3_/CNT‐coated fabric demonstrated precise and stable strain detection across various body motions, confirming its suitability as a multifunctional, wearable strain sensor for bio‐medical monitoring.

### 4.6 Advanced Applications: Emergency Morse Code Signaling and Safety‐Line Alert System

Beyond conventional strain sensing, the multifunctional MXene/ZnO NR:WO_3_/CNT‐coated cotton fabric was further explored for its real‐time interactive signaling capabilities, particularly for use in emergency communication and safety systems.

#### 4.6.1 Morse Code Signaling via Finger Motion

**FIGURE 8:**
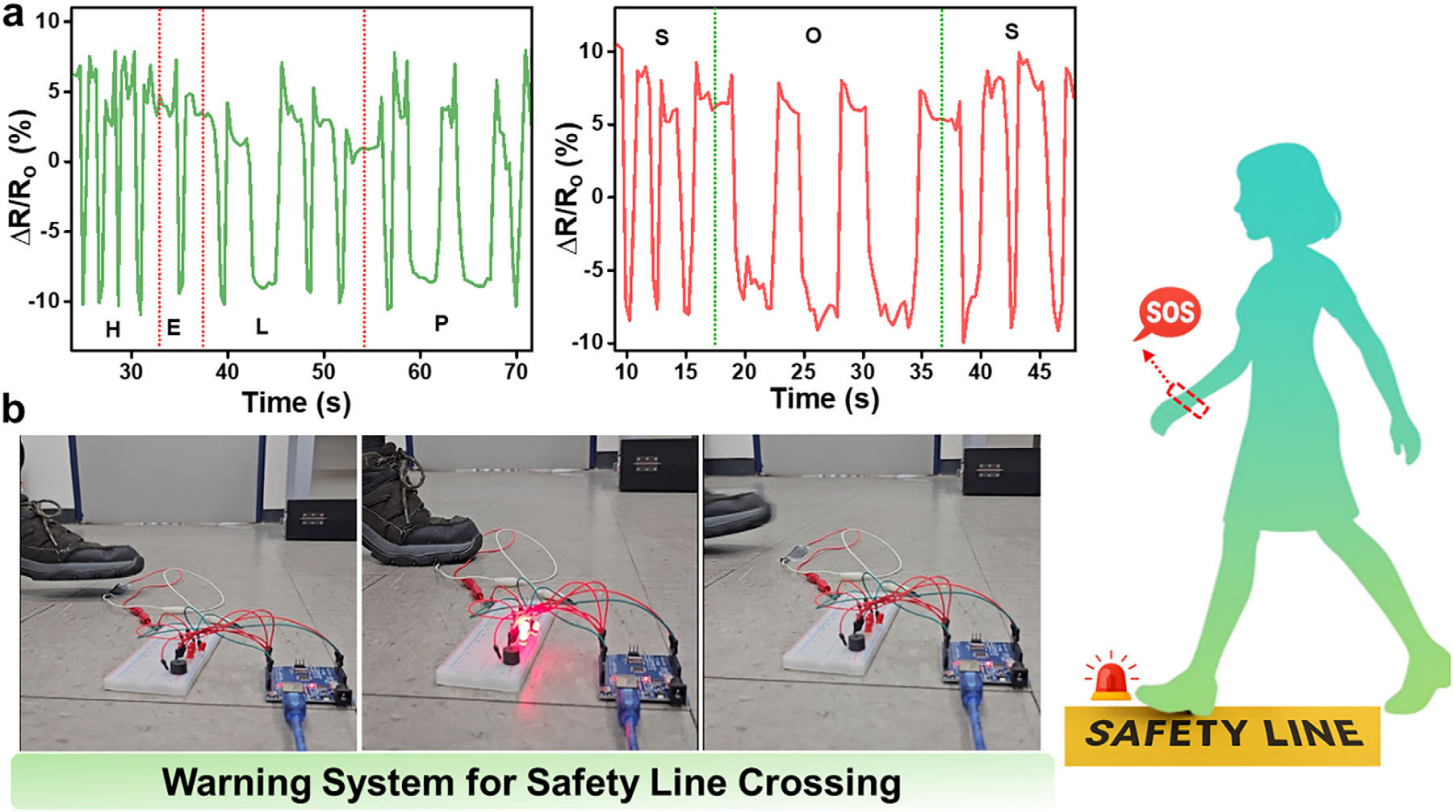
(a) Morse code signaling demonstration of MXene/ZnO NRs:WO_3_/CNT‐coated cotton fabric for emergency signaling. (b) Photographs of MXene/ZnO NRs:WO_3_/CNT‐coated cotton fabric used as a warning system for safety‐line crossing.

To demonstrate its potential as a wearable emergency signaling interface, the fabric sensor was mounted onto the index finger of a user and utilized to simulate Morse code transmission. By intentionally varying the duration and interval of finger bends corresponding to “dot” (short press) and “dash” (long press) signals distinct and time‐resolved resistance changes were observed in the sensor output. The shorter bending durations produced rapid resistance spikes (dots), and prolonged bending yielded broader signal profiles (dashes), thereby allowing binary encoding of Morse signals. The Morse code signaling capability of the MXene/ZnO NRs:WO_3_/CNT‐coated cotton fabric is demonstrated in Figure 8a. This responsive behavior demonstrates the capacity of the sensor to transmit critical information in a hands‐free, low‐resource emergency scenario, such as in confined or hazardous environments where vocal or electronic communication may be compromised.

#### 4.6.2 Safety‐Line Crossing Alert System

Furthermore, the multifunctional fabric was applied as a safety alert sensor for restricted zone monitoring. The fabric was integrated along a designated safety line, and the resistance‐based response was monitored when mechanical pressure (e.g., footstep) was applied. Upon stepping directly on the fabric while crossing the line, mechanical compression stimulus is applied, which deformed the trilayer sensor. This disrupted and modulated its conductive pathways and triggered a significant and immediate change in resistance, which was pre‐calibrated to activate an external visual or audible warning signal using an interfaced microcontroller (Arduino) (Figure 8b; video S2). The high sensitivity, short response time, and high mechanical durability under repetitive loading make the fabric a promising candidate for application in real‐time alert systems in industrial or hazardous environments.

**FIGURE 9:**
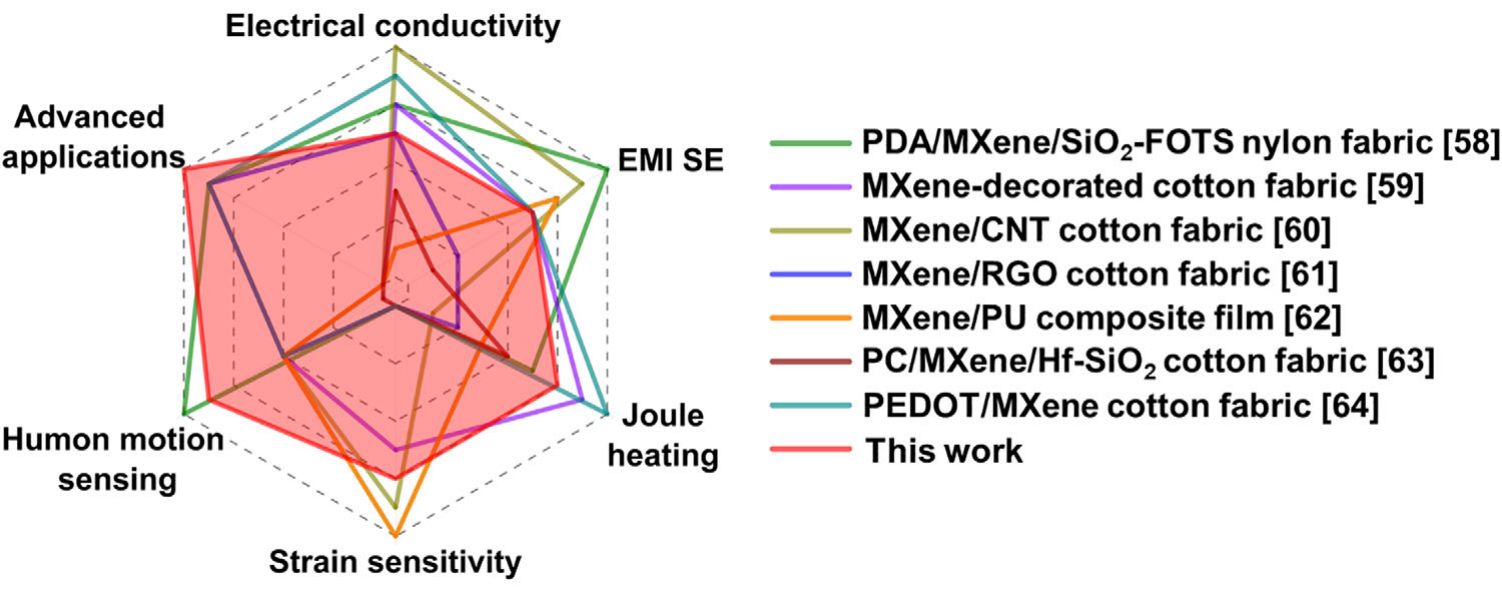
Radar chart comparing the multifunctional performance of the MXene/ZnO NRs:WO_3_/CNT‐coated cotton fabric with representative MXene‐based multifunctional textile systems reported in the literature.

Further, to benchmark the proposed trilayer‐coated cotton fabric against representative MXene‐based multifunctional textiles reported in the literature, Figure 9 presents a radar chart comparison [58, 59, 60, 61, 62, 63, 64]. While certain designs exhibit excellence in specific metrics such as the ultra‐high GF of MXene/PU composite films [54] or the extreme Joule heating of PEDOT/MXene fabrics [56], these systems typically compromise other key functionalities or lack application versatility. In contrast, the proposed trilayer architecture delivers well‐balanced, high‐level performance across multiple domains, including a gauge factor of 0.47, rapid response (∼200 ms) for human motion sensing, EMI shielding effectiveness of 34.4 dB, and Joule heating reaching 110°C within ∼15 s at only 3.5 V.

The cytocompatibility of the MXene/ZnO:WO_3_/CNT‐coated cotton fabric was assessed based on reported literature for each constituent material (Table S4). Cotton is inherently skin‐friendly, while ZnO and WO_3_ have been widely reported to exhibit low cytotoxicity at relevant concentrations. Although CNTs and MXenes can show dose‐dependent cytotoxicity in their free form, studies indicate that their immobilization within composite significantly mitigate biological exposure. Therefore, considering the immobilized multilayer architecture and literature reports, the coated cotton fabric is expected to exhibit acceptable cytocompatibility for wearable applications.

**FIGURE 10:**
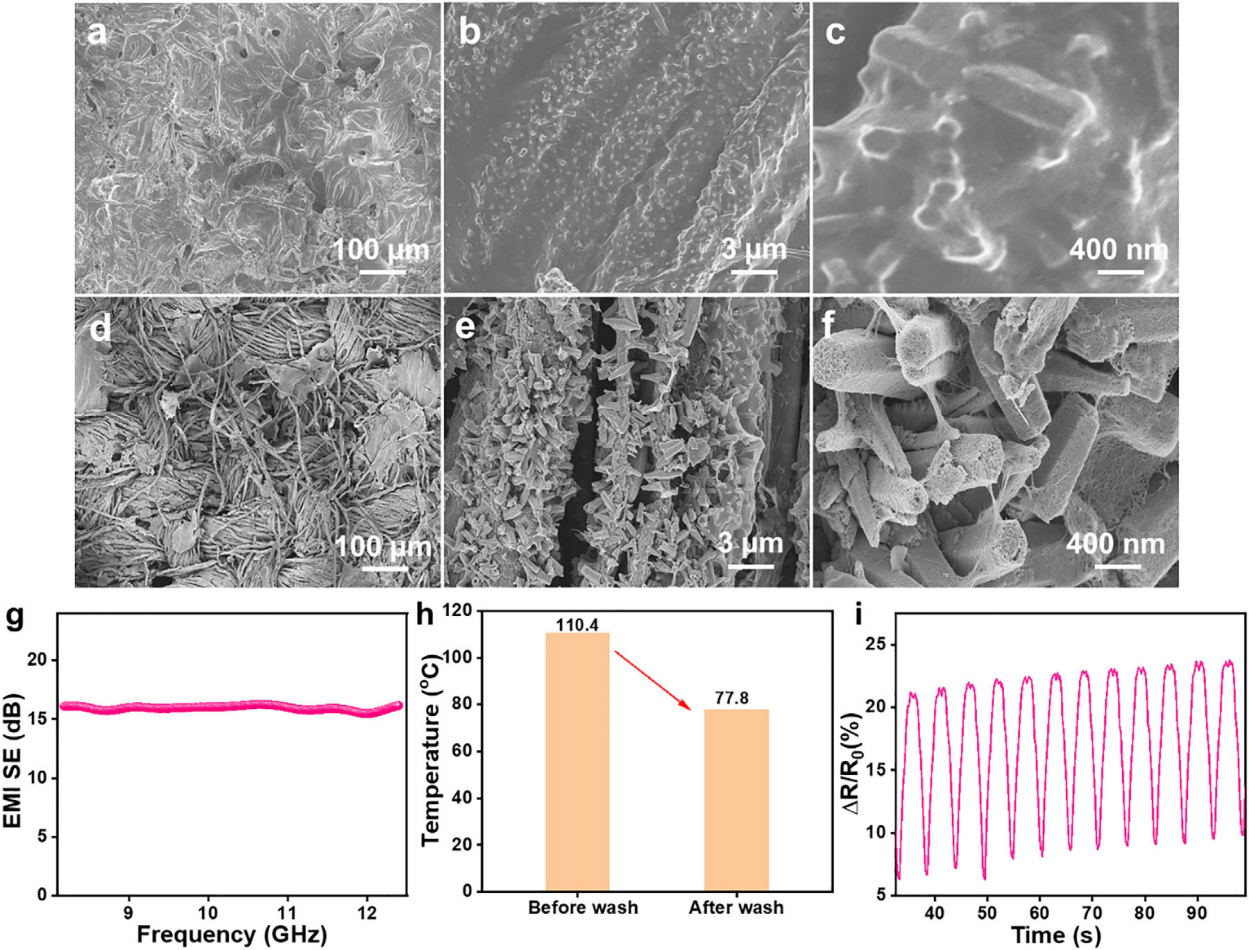
Wash durability of the MXene/ZnO NR: WO_3_/CNT‐coated cotton fabric. (a–c) FE‐SEM images of the MXene/ZnO NR: WO_3_/CNT coating before washing. (d–f) FE‐SEM images of the MXene/ZnO NR: WO_3_/CNT coating after 10 washing cycles. (g) EMI shielding effectiveness after washing. (h) Joule heating performance at an applied voltage of 3.5 V for ∼15 s after washing. (i) Strain‐sensing performance of the MXene/ZnO NR: WO_3_/CNT‐coated cotton fabric at 25% strain after 10 washing cycles.

Importantly, the robustness of the multifunctional MXene/ZnO NRs:WO_3_/CNT coating was further evaluated through wash‐fastness testing to assess its practical applicability in wearable textiles. The coated cotton fabric was subjected to vigorous washing under commercial detergent solution at 1500 rpm for 30 min per cycle, followed by ambient drying. After 10 washing cycles, the fabric retained electrical conductivity, although the sheet resistance increased moderately to ∼285 Ω, indicating partial degradation of the conductive network while preserving percolation pathways. To elucidate the origin of the performance changes after washing, surface morphology was examined by FE‐SEM (Figure 10a–f). The images reveal that the outer CNT layer was partially removed from the fiber surface after repeated washing, which is expected under strong mechanical agitation. Notably, CNTs interlocked within the ZnO NRs:WO_3_ framework remained firmly anchored, forming a spider‐web‐like conductive network bridging adjacent nanorods. This morphology confirms that the ZnO NRs:WO_3_ interlayer plays a critical mechanical reinforcement role, suppressing complete delamination of conductive fillers and maintaining residual electrical connectivity. Correspondingly, functional performance exhibited a moderate decline after washing. The EMI shielding effectiveness decreased to ∼16 dB (Figure 10g), and the maximum Joule heating temperature was reduced to ∼77°C (Figure 10h) and good strain sensing performance (Figure 10i) under identical operating conditions. These reductions are primarily attributed to the partial loss of the outer CNT layer, which weakens surface conductivity, conduction loss, and interfacial polarization pathways that are essential for efficient electromagnetic attenuation and electrothermal conversion. Nevertheless, the retention of CNTs embedded within the ZnO NRs:WO_3_ interlayer enables the fabric to preserve its multifunctional response, albeit at a reduced level. Further, the cotton substrate itself remained structurally intact and mechanically stable throughout the washing cycles, underscoring its suitability as a flexible and resilient wearable platform. Despite some performance attenuation, the coated fabric maintains its multifunctional character after repeated laundering, demonstrating that the MXene/ZnO NRs:WO_3_/CNT architecture possesses acceptable wash durability for practical wearable applications. Further optimization of CNT anchoring strategies and interfacial binding is expected to enhance long‐term wash stability without compromising multifunctional performance.

This synergy of strain sensitivity, electrical conductivity, and electrothermal capability, combined with mechanical durability under cyclic loading, unlocks niche real‐world applications such as emergency Morse‐code signalling and safety‐line alert systems, where rapid strain detection and stable electrical output are critical in hazardous environments. Furthermore, its strong EMI attenuation and rapid heating extend the utility of the fabric to wearable thermal management, EMI protection for commercial electronics, and de‐icing under extreme conditions, surpassing conventional single‐ or dual‐functional textile solutions. Wash‐fastness evaluation further demonstrates the practical robustness of the trilayer architecture: despite partial removal of the outer CNT layer after repeated laundering, the coated fabric retained electrical conductivity and multifunctional performance, supported by CNTs anchored within the ZnO NRs:WO_3_ interlayer. This residual conductive network preserves key functionalities even under realistic washing conditions. Collectively, these results establish the MXene/ZnO NRs:WO_3_/CNT‐coated cotton as a robust and adaptable platform for next‐generation intelligent wearable systems, balancing high multifunctional performance with essential durability requirements for real‐world use.

## 5 Conclusion

This study successfully fabricated a multifunctional, lightweight, and breathable smart textile by applying a sandwich‐structured coating, comprising conductive MXene, semiconductive ZnO NRs doped with WO_3_, and highly conductive CNTs, onto a cotton fabric. Each component was deliberately selected: MXene provides high conductivity but suffers from restacking and oxidation; ZnO:WO_3_ acts as a semiconductive spacer that stabilizes MXene and introduces defect‐mediated functionalities; and CNTs form a robust percolation network, enhancing surface conductivity and protecting underlying layers. This hierarchical structure not only overcomes the limitations of conventional conductive‐only materials but also leverages the synergistic interplay between conductive and semiconductive nanostructures to achieve enhanced multifunctional performance. The coated fabric exhibited excellent strain‐sensing behavior, with rapid response and recovery times, and maintained stable resistance under varying stretching rates. Moreover, the fabric sensor responded reliably to diverse mechanical deformations such as bending, twisting, and pressing, confirming its potential for human motion monitoring. As a wearable thermal heater, the fabric demonstrated rapid and uniform Joule heating, reaching approximately 110°C within ∼15 s, and retained consistent performance over multiple on/off cycles. Furthermore, the fabric exhibited excellent EMI SE of approximately 34.4 dB in the X‐band, which increased to approximately 78 dB with increasing fabric thickness, surpassing commercial thresholds for practical applications. Despite the trilayered coatings, the textile retained its lightweight and breathable properties.

Beyond fundamental characterization, the real‐world application of sandwich‐structured coatings onto a cotton fabric was successfully demonstrated. Specifically, the multifunctional textile enabled emergency signaling during critical events and safety alert triggering when a user crossed a designated safety boundary. Thus, the trilayer design is not redundant but represents a rational strategy in which each layer fulfills a distinct role, and together they deliver multifunctionality that would not be achievable in single‐ or dual‐component systems. Overall, this study introduces a novel strategy for developing high‐performance, multifunctional wearable textiles suitable for next‐generation smart electronics, with promising applications in personal safety, communication, and health monitoring.

## Author Contributions

Thirumalaisamy Suryaprabha: Writing – original draft, Visualization, Validation, Methodology, Investigation, Formal analysis, Data curation, Conceptualization. Chunghyeon Choi: Visualization, Resources, Investigation, Formal analysis, Data curation. Sujith Lal: Software, Resources, Investigation, Data curation. Min‐Young Seo: Investigation, Data curation. Tae‐Wook Kim: Investigation, Resources. Ergang Wang: Writing – review & editing, Visualization, Validation. Byungil Hwang: Writing – review & editing, Visualization, Validation, Supervision, Resources, Project administration, Methodology, Funding acquisition, Conceptualization.

## Funding

This work was financially supported by the Global Research Development Center (GRDC) Cooperative Hub Program through the National Research Foundation of Korea (NRF) funded by the Ministry of Science and ICT (MSIT) (RS‐2023‐00257595).

## Conflicts of Interest

The authors declare no conflicts of interest.

## Supporting information




**Supporting File 1**: advs74855‐sup‐0001‐SuppMat.docx.

**Supporting File 2**: advs74855‐sup‐0002‐VideoS1.pptx.

## Data Availability

The data that support the findings of this study are available from the corresponding author upon reasonable request.
