# Supplementary material for: Hierarchical MXene/ZnO Nanorods: WO3/CNT Trilayer Coatings on Cotton for High‐Performance Multifunctional Wearable Fabrics
Source: Adv Sci (Weinh). 2026 Mar 18;13(25):e21175. doi: 10.1002/advs.202521175 (PMC13137848; doi:10.1002/advs.202521175)
Supplement: Supplementary file 1 — Supporting File 1: advs74855‐sup‐0001‐SuppMat.docx. [file ADVS-13-e21175-s001.docx]

**Supporting information**

**Hierarchical MXene/ZnO nanorods: WO₃/CNT Trilayer Coatings on Cotton for High-Performance Multifunctional Wearable Fabrics**

*Thirumalaisamy Suryaprabha^a^, Chunghyeon Choi^a^, Sujith Lal^b^, Ergang Wang^c*^,* *Byungil Hwang^b*^*

*^a^Department of Intelligent Semiconductor Engineering, Chung-Ang University, Seoul, 06974, Republic of Korea*

*^b^School of Integrative Engineering, Chung-Ang University, Seoul, 06974, Republic of Korea*

*^c^Department of Chemistry and Chemical Engineering, Chalmers University of Technology, Göteborg, SE‑412 96 Sweden*

**Corresponding authors:* [*bihwang@cau.ac.kr*](mailto:bihwang@cau.ac.kr)*; ergang@chalmers.se*

**Characterization of MXene**

**
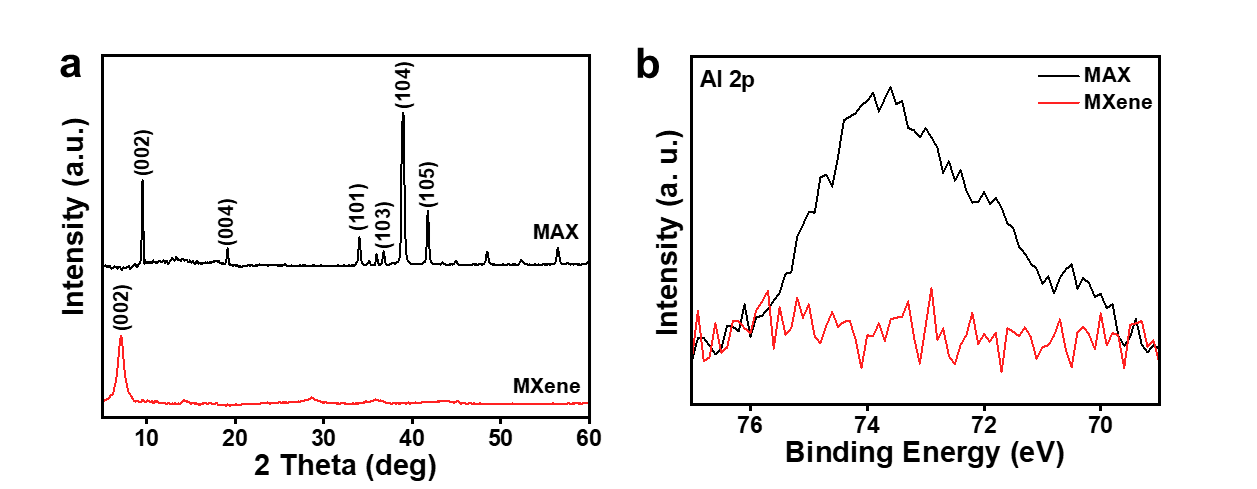
**

**Fig. S1** **a** XRD patterns of MAX phase and MXene before and after HF etching; **b** Al 2p XPS spectra of MAX and etched MXene.

Figure S1a presents the XRD patterns of the pristine MAX phase and the corresponding MXene obtained after HF etching. The MAX phase exhibits well-defined diffraction peaks. After HF treatment, the intensities of these characteristic MAX peaks completely disappeared. Meanwhile, the (002) diffraction peak corresponding to MXene shows an obvious shift toward lower diffraction angles after etching. This shift, together with the suppression of MAX-related reflections, confirms the successful transformation of MAX into MXene. To further verify the selective etching of the Al layer, XPS analysis was performed (Fig. S1b). The XPS spectrum of the pristine MAX phase displays a distinct Al 2p signal, which is a characteristic feature of the Al-containing MAX structure. After HF etching, the Al 2p peak is no longer detected in the MXene sample, demonstrating the complete removal of Al from the parent MAX phase. This result provides direct chemical evidence for the effectiveness of the HF etching process and corroborates the XRD observations. Taken together, the combined XRD and XPS analyses unambiguously confirm the successful synthesis of MXene.

**Characterization of WO_3_**


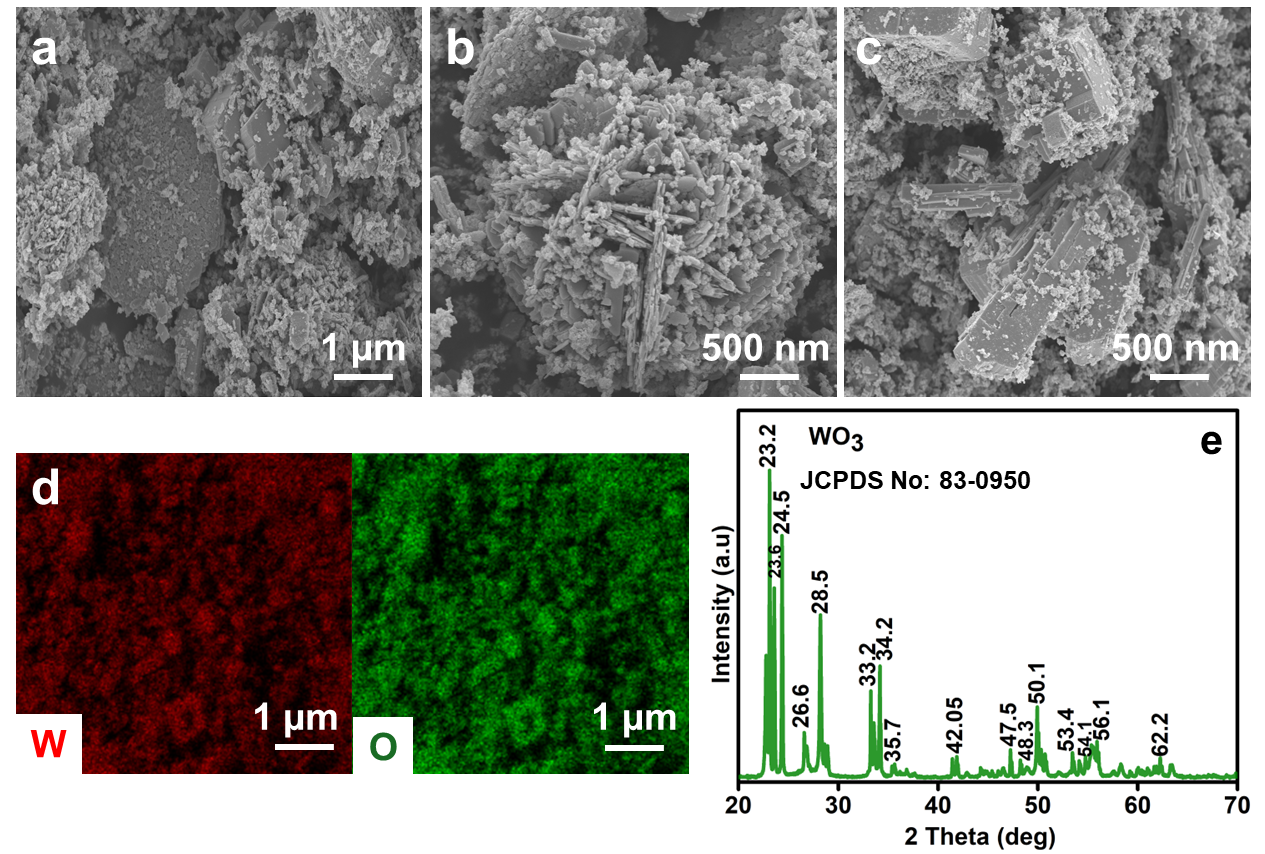


**Fig. S2** **a-c** FE-SEM images of WO_3_; **d** Elemental mapping images of WO_3_; **e** XRD pattern of WO_3_.

The morphological, elemental, and structural characteristics of the synthesized WO₃ were investigated using SEM imaging, EDX mapping, and XRD analyses. SEM images revealed that the WO₃ nanostructures exhibited a well-defined, cube and plate-like mixed morphology with sharp edges. This suggests a large surface area and high crystalline order, which are favorable for enhancing interfacial interactions in hybrid coatings. EDX mapping confirmed the stoichiometric formation of WO₃. Furthermore, XRD analysis demonstrated the crystalline nature of the synthesized material. The diffraction peaks were consistent with those of monoclinic WO₃ (JCPDS No: 83-0950), confirming phase purity and the absence of secondary phases. The strong intensity and sharpness of the peaks further supported the high crystallinity of the material. Overall, these findings confirm the successful fabrication of highly crystalline and uniform WO₃ nanostructures, which provide a promising foundation for their integration into multifunctional textile applications.


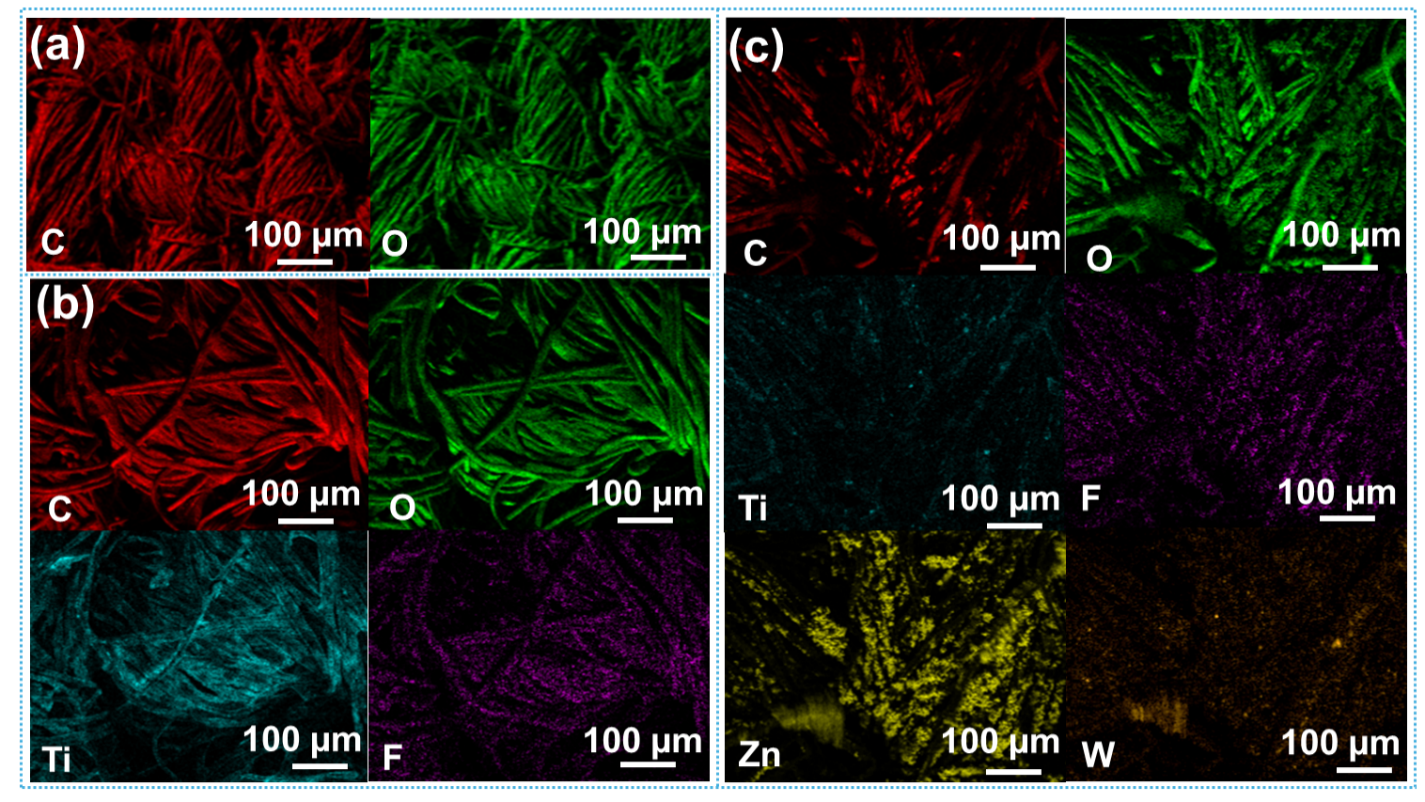


**Fig. S3** EDAX mapping of **a** normal cotton, **b** MXene-coated cotton, and **c** MXene-ZnO NRs:WO_3_-coated cotton


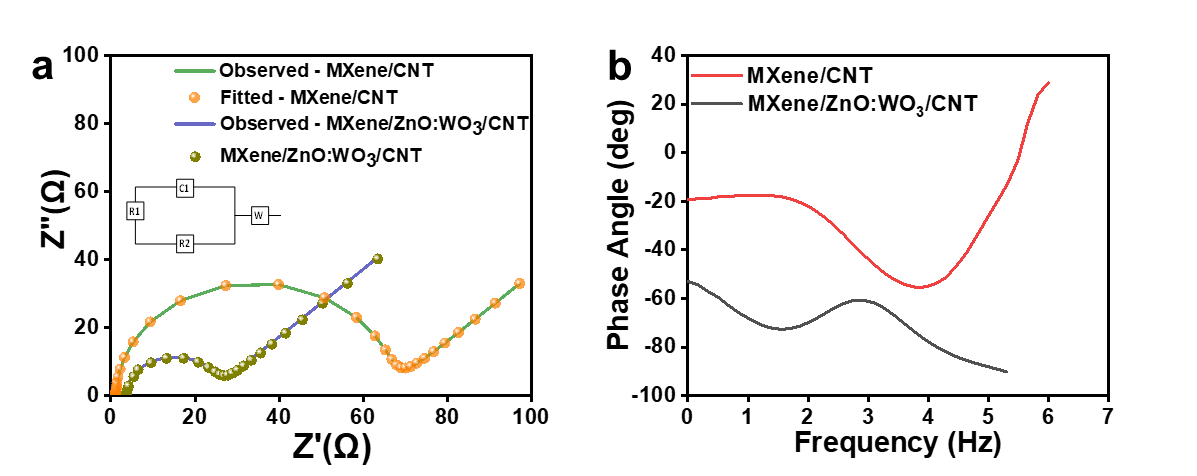


**Fig. S4** Impedance analysis: **a** Nyquist and **b** Bode plots of MXene/CNT and MXene/ZnO:WO_3_/CNT cotton samples


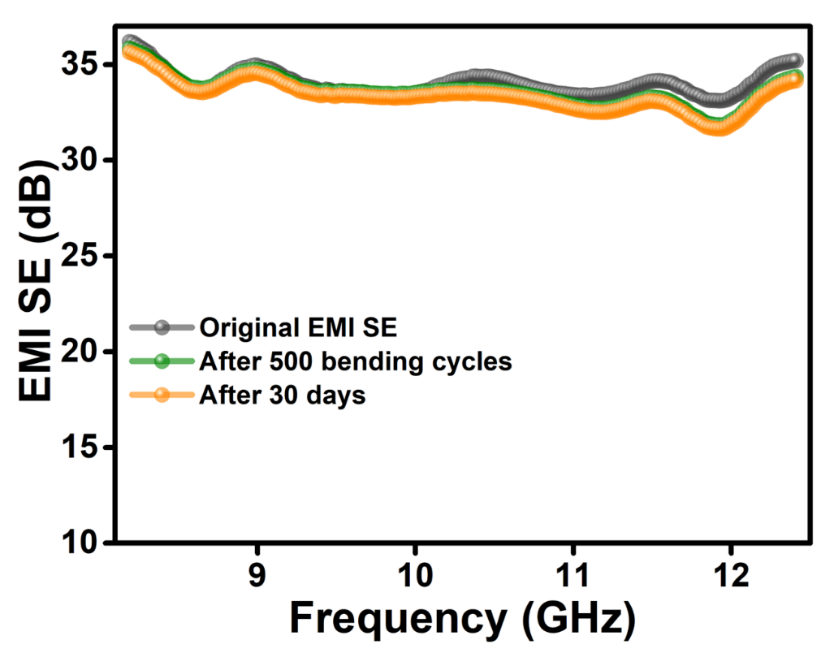


**Fig. S5** Mechanical durability of electromagnetic interference shielding effectiveness (EMI SE) of the MXene/ZnO:WO₃/CNT-coated cotton fabric


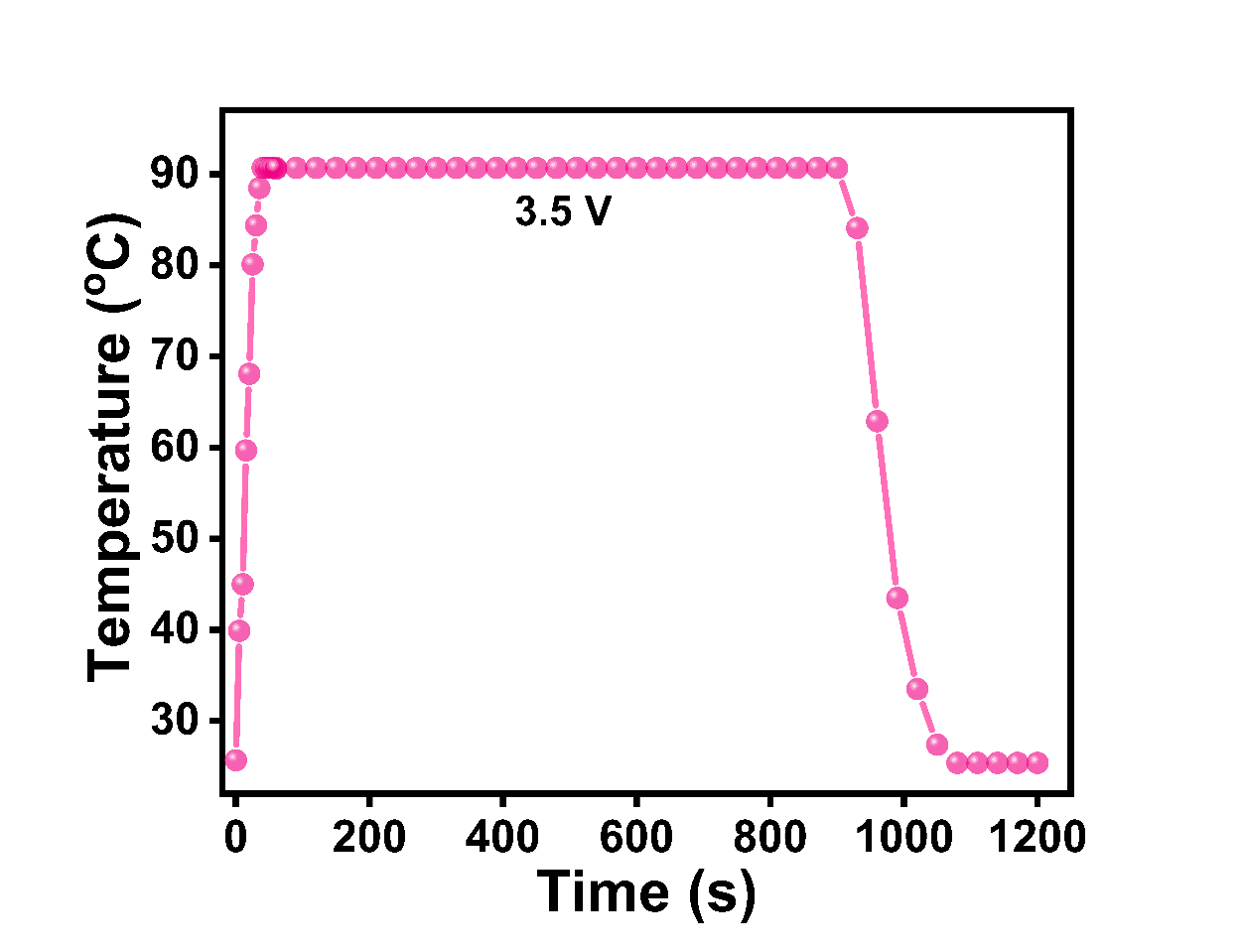


**Fig. S6** Joule heating performance of the MXene/CNT-coated cotton fabric at applied voltage of 3.5 V.


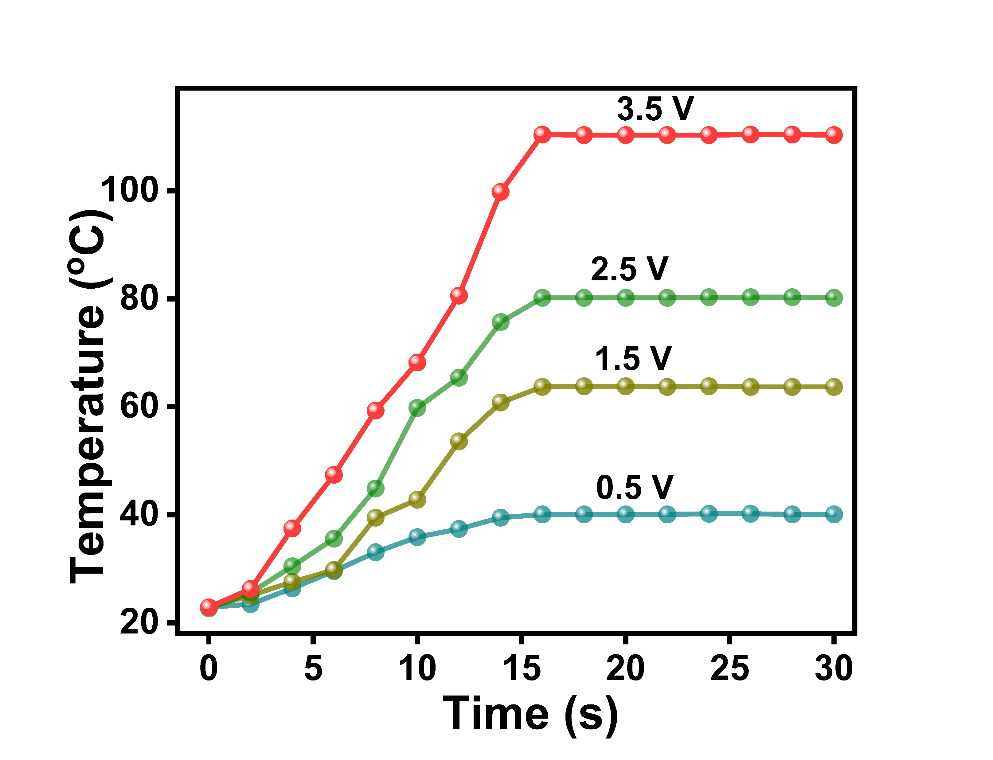


**Fig. S7** Joule heating performance of the MXene/ZnO:WO₃/CNT-coated cotton fabric during the initial 30 s under applied voltage.


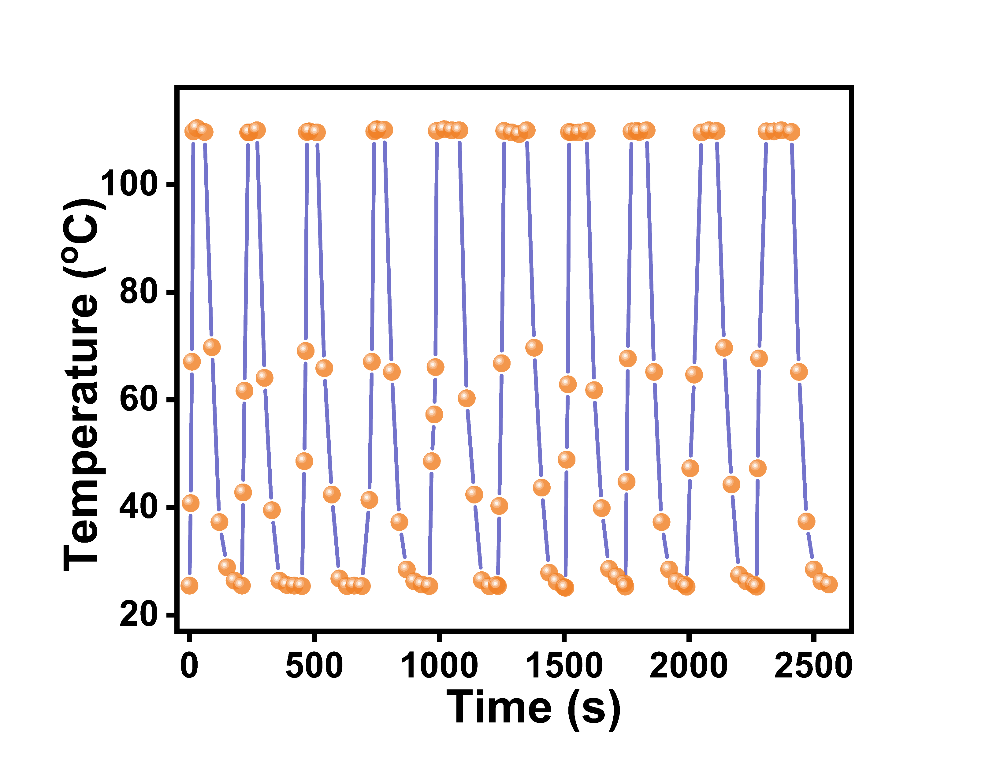


**Fig. S8** Mechanical durability of joule heating performance of the MXene/ZnO:WO₃/CNT-coated cotton fabric after 500 bending cycles.


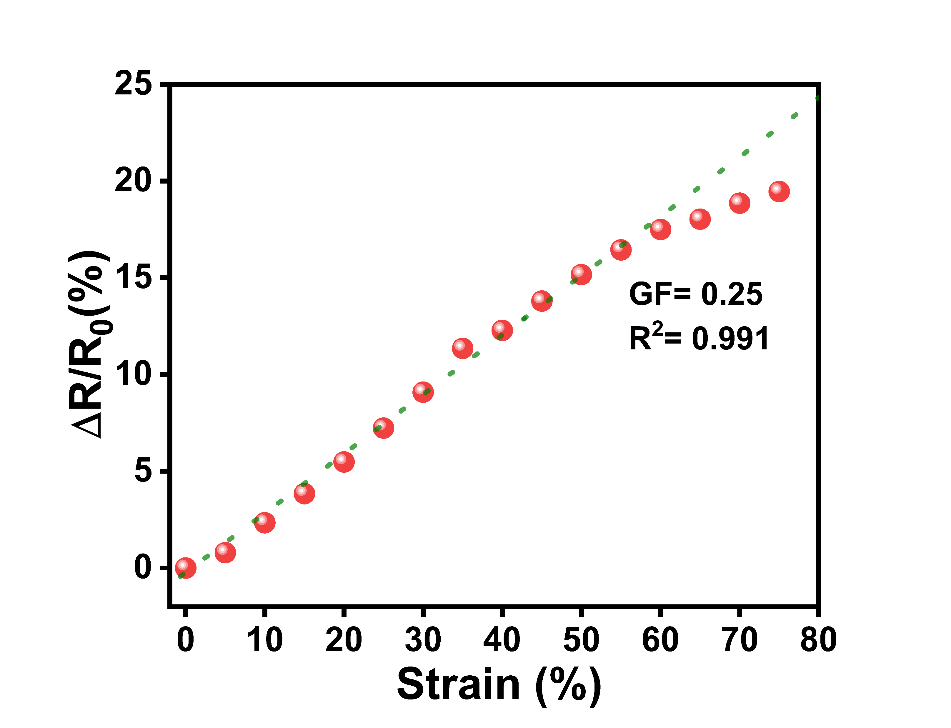


**Fig. S9** Relative resistance change of MXene/CNT cotton under different strain levels

**Table S1.** Comparison of EMI performance of the proposed trilayer-coated cotton fabric with previously published works

| **Functional materials/Thickness** | **Conductivity** | **Frequency band** | **SE_T_ (**dB) | **Ref** |
| --- | --- | --- | --- | --- |
| PVA/natural rubber latex cotton fabric (0.20 mm) | ~1 kΩ/cm^2^ | 8.2–12.4 GHz | 37.7 | [1] |
| PEDOT/Cu/Ag fabric (20 µm) | 0.8 Ω/sq | 100 MHz to 13.6 GHz | ~45 | [2] |
| PDA/MXene/SiO_2_-FOTS nylon fabric (0.8 mm) | 5.1 Ω/sq | 8.2–12.4 GHz | 66.5 | [3] |
| MXene-decorated cotton woven fabric (0.33 mm) | 5 Ω/sq | 8.2–12.4 GHz | 36 | [4] |
| MXene-modified aramid nonwoven fabric (1 mm) | 4.36 Ω/sq | 8.2–12.4 GHz | 35 | [5] |
| MXene/AgNW silk fabric (120 µm) | 0.8 Ω/sq | 8.2–12.4 GHz | ~ 43 | [6] |
| MXene-cotton fabric (0.28 mm) | 0.6 Ω/cm | 8.2–12.4 GHz | ~ 80 | [7] |
| AgNWs/fibroin degummed silk composite fibers (NA) | 8.8 Ω/cm | 8.2–12.4 GHz | 38 | [8] |
| Thiol-modified rGO-WPU/cotton (1 mm) | ~5 K Ω/sq | 8.2–12.4 GHz | 30.2 | [9] |
| MXene/CNT cotton knitted fabric (138 µm) | 0.008 Ω/sq | 8.2–12.4 GHz | 46 | [10] |
| MXene/RGO cotton fabric (553±61 nm) | ~15 Ω/sq | 8.2–12.4 GHz | 29 | [11] |
| MXene/PU composite film (600 µm) | ~97 Ω/sq | 8.2–12.4 GHz | 38 | [12] |
| PC/MXene/Hf-SiO_2_ film (NA) | 35.1 Ω/sq | 8.2–12.4 GHz | 22.5 | [13] |
| PEDOT/MXene cotton fabric (NA) | 3.6 Ω/sq | 8.2–12.4 GHz | 36.6 | [14] |
| MXene/ZnO NR:WO_3_/CNT-coated cotton fabric (0.21 mm) | 15 Ω/sq | 8.2–12.4 GHz | 34.4 | This work |

**Table S2.** Comparison of Joule heating performance of the proposed trilayer-coated cotton fabric with previously published works

| **Materials/Thickness** | **Electrical conductivity** | **Applied voltage** | **Joule Heating Temperature** | **Ref** |
| --- | --- | --- | --- | --- |
| MXene decorated fabric (0.62 mm) | 2.2 Ω/sq | 5 V | 68.4°C after 25 s | [15] |
| AgNPs/MXene/Cotton yarn (NA) | 27 Ω/cm | 6 V | 92.4°C | [16] |
| CNT modified fabric (NA) | 5 kΩ | 40 V | 50°C after ~150 s | [17] |
| PANI/PEDOT: PSS cotton (NA) | 1.39 kΩ/cm | 15 V | 38°C after 90 s | [18] |
| PVDF-AgNW/MXene film (NA) | 0.9 Ω/sq | 2.5 V | 77°C after ~25 s | [19] |
| PPy/SWCNT/PDA/Cotton (NA) | 8 Ω/sq | 5 V | 144.6°C after 100 s | [20] |
| PEDOT/MXene cotton (NA) | 3.6 Ω/sq | 12 V | 193.1°C after ~45 s | [14] |
| rGO/PEDOT:PSS textile (NA) | 153 Ω/sq | 10 V | 70°C after 30 s | [21] |
| PDA/MXene/SiO_2_-FOTS nylon fabric (0.8 mm) | 5.1 Ω/sq | 8 V | 102.3°C after 100s | [3] |
| MXene-decorated cotton woven fabric (0.33 mm) | 5 Ω/sq | 6 V | 150°C after 50s | [4] |
| AuNP/DWCNT textile (NA) | 806 Ω | 9 V | 59°C after ~70 s | [22] |
| MXene/CNT cotton knitted fabric (138 µm) | 0.008 Ω/sq | 2 V | 46.6°C after 10s | [10] |
| MXene/RGO cotton fabric (553±61 nm) | ~15 Ω/sq | 12 V | 66.7°C after ~50s | [11] |
| MXene/PU composite film (600 µm) | ~97 Ω/sq | 6 V | 85.8°C after 12s | [12] |
| PC/MXene/Hf-SiO_2_ film (NA) | 35.1 Ω/sq | 13 V | 100°C after 15s | [13] |
| PEDOT/MXene cotton fabric (NA) | 3.6 Ω/sq | 12 V | 193.1°C after 50s | [14] |
| MXene/ZnO NR:WO_3_/CNT-coated cotton fabric (0.21 mm) | 15 Ω/sq | 3.5 V | 110°C after 15 s | This work |

**Table S3.** Comparison of strain sensing performance of the proposed trilayer-coated cotton fabric with previously published works

| **Materials/Thickness** | **Electrical conductivity** | **Gauge Factor** | **Response/recovery time** | **Ref** |
| --- | --- | --- | --- | --- |
| Graphite-PU/CSK band (NA) | 25-27 KΩ | 26.8 | Not specified | [23] |
| Polyester/PU/GO fiber (0.4 mm) | 0.136 S m^-1^ | 10 | <100 ms | [24] |
| PU/CNT yarn (NA) | 8.77 S/cm | 1.7 | Not specified | [25] |
| MXene/PPy textile (NA) | 93.33 S/m | ~3.18 | Not specified | [26] |
| Carbon/graphene/ecoflex substrate (0.75 mm) | 120.9 k Ω | 35 V | ~200 ms | [27] |
| AgNWs/MXene (0.636 mm) | Not specified | 2.67 | 735/717 ms | [28] |
| MXene-CNTs/TPU/Ecoflex conductive composites (4.77 ± 0.86 μm) | Not specified | 1719.2 | 50 ms | [29] |
| CNT/MXene@TPU flexible sensor (NA) | ~680 Ω/cm | 30.5 | 57 ms | [21] |
| PDA/MXene/SiO_2_-FOTS nylon fabric (NA) | 5.1 Ω/sq | 9.5 | 60-90 ms | [3] |
| MXene/ZnO NR:WO_3_/CNT-coated cotton fabric (0.21 mm) | 15 Ω/sq | 0.47 | 200-210 ms | This work |

**Table S4.** Overview of literature-reported cytotoxicity and biocompatibility of individual materials employed in this work for wearable applications.

| **Material** | **Reported cytotoxicity / biocompatibility test** | **Application context** | **Reference** |
| --- | --- | --- | --- |
| Cotton | Skin-friendly / biocompatible | Wearable fabrics | [30] |
| MXene | Dose-dependent cytotoxicity (limited reports) | Flexible and wearable electronics; in vitro / in vivo studies | [31-33] |
| ZnO | Low cytotoxicity (dose-dependent) | Antimicrobial and UV-protective textiles | [34, 35] |
| WO₃ | Low cytotoxicity | Gas-sensing, biomedical and wearable applications | [36, 37] |
| MWCNTs | Dose- and surface-dependent cytotoxicity | Wearable composites | [38, 39] |

**References:**

[1] S. Ghosh, S. Remanan, S. Mondal, S. Ganguly, P. Das, N. Singha, N.C. Das (2018) An approach to prepare mechanically robust full IPN strengthened conductive cotton fabric for high strain tolerant electromagnetic interference shielding, Chem Eng J 344: 138-154. <https://doi.org/10.1016/j.cej.2018.03.039>.

[2] S. Riaz, S. Naz, A. Younus, A. Javid, S. Akram, A. Nosheen, M. Ashraf (2022) Layer by layer deposition of PEDOT, silver and copper to develop durable, flexible, and EMI shielding and antibacterial textiles, Colloids Surf Physicochem Eng Aspects 650: 129486. <https://doi.org/10.1016/j.colsurfa.2022.129486>.

[3] J. Peng, H. Cheng, J. Liu, W. Han, T. Wu, Y. Yin, C. Wang (2023) Superhydrophobic MXene-based fabric with electromagnetic interference shielding and thermal management ability for flexible sensors, Adv Fiber Mater 5: 2099-2113. <https://doi.org/10.1007/s42765-023-00328-x>.

[4] X. Zhang, X. Wang, Z. Lei, L. Wang, M. Tian, S. Zhu, H. Xiao, X. Tang, L. Qu (2020) Flexible MXene-decorated fabric with interwoven conductive networks for integrated joule heating, electromagnetic interference shielding, and strain sensing performances, ACS Appl Mater Interfaces 12: 14459-14467. <https://doi.org/10.1021/acsami.0c01182>.

[5] X. Wang, Z. Lei, X. Ma, G. He, T. Xu, J. Tan, L. Wang, X. Zhang, L. Qu, X. Zhang (2022) A lightweight MXene-Coated nonwoven fabric with excellent flame retardancy, emi shielding, and electrothermal/photothermal conversion for wearable heater, Chem Eng J 430: 132605. <https://doi.org/10.1016/j.cej.2021.132605>.

[6] L.X. Liu, W. Chen, H.B. Zhang, Q.W. Wang, F. Guan, Z.Z. Yu (2019) Flexible and multifunctional silk textiles with biomimetic leaf‐like MXene/silver nanowire nanostructures for electromagnetic interference shielding, humidity monitoring, and self‐derived hydrophobicity, Adv Funct Mater 29: 1905197. <https://doi.org/10.1002/adfm.201905197>.

[7] S. Uzun, M. Han, C.J. Strobel, K. Hantanasirisakul, A. Goad, G. Dion, Y. Gogotsi (2021) Highly conductive and scalable Ti_3_C_2_T_x_ -coated fabrics for efficient electromagnetic interference shielding, Carbon 174: 382-389. <https://doi.org/10.1016/j.carbon.2020.12.021>.

[8] Y. Lu, J. Xu, Y. Liu, J. Ban, X. Li, M. Li, Y. Zhou, D. Wang, L. Piao (2024) Capillarity-assisted assembly of composite fibers to enable highly conductive fabrics for electromagnetic interference shielding, Composites Science and Technology 253: 110659. <https://doi.org/10.1016/j.compscitech.2024.110659>.

[9] Y. Wang, W. Wang, R. Xu, M. Zhu, D. Yu (2019) Flexible, durable and thermal conducting thiol-modified rGO-WPU/cotton fabric for robust electromagnetic interference shielding, Chem Eng J 360: 817-828. <https://doi.org/10.1016/j.cej.2018.12.045>.

[10] C. Xie, Y. Wang, W. Wang, D. Yu (2022) Flexible, conductive and multifunctional cotton fabric with surface wrinkled MXene/CNTs microstructure for electromagnetic interference shielding, Colloids Surf Physicochem Eng Aspects 651: 129713. <https://doi.org/10.1016/j.colsurfa.2022.129713>.

[11] X. Zheng, W. Nie, Q. Hu, X. Wang, Z. Wang, L. Zou, X. Hong, H. Yang, J. Shen, C. Li (2021) Multifunctional RGO/Ti_3_C_2_T_x_ MXene fabrics for electrochemical energy storage, electromagnetic interference shielding, electrothermal and human motion detection, Mater Des 200: 109442. <https://doi.org/10.1016/j.matdes.2020.109442>.

[12] X. Li, M. Yang, W. Qin, C. Gu, L. Feng, Z. Tian, H. Qiao, J. Chen, J. Chen, S. Yin (2023) MXene-based multilayered flexible strain sensor integrating electromagnetic shielding and Joule heat, Colloids Surf Physicochem Eng Aspects 658: 130706. <https://doi.org/10.1016/j.colsurfa.2022.130706>.

[13] B. Zhou, Z. Li, Y. Li, X. Liu, J. Ma, Y. Feng, D. Zhang, C. He, C. Liu, C. Shen (2021) Flexible hydrophobic 2D Ti_3_C_2_T_x_-based transparent conductive film with multifunctional self-cleaning, electromagnetic interference shielding and joule heating capacities, Compos Sci Technol 201: 108531. <https://doi.org/10.1016/j.compscitech.2020.108531>.

[14] X. Zheng, J. Shen, Q. Hu, W. Nie, Z. Wang, L. Zou, C. Li (2021) Vapor phase polymerized conducting polymer/MXene textiles for wearable electronics, Nanoscale 13: 1832-1841. <https://doi.org/10.1039/d0nr07433k>.

[15] X. Zheng, P. Wang, X. Zhang, Q. Hu, Z. Wang, W. Nie, L. Zou, C. Li, X. Han (2022) Breathable, durable and bark-shaped MXene/textiles for high-performance wearable pressure sensors, EMI shielding and heat physiotherapy, Compos Part A- Appl Sci Manuf 152:  106700. <https://doi.org/10.1016/j.compositesa.2021.106700>.

[16] G. Hu, H. Zhao, N. Zhong, H. Zhao, H. Zhang, A. Zang, F. Kong, J. Hu (2023) Highly conductive cellulose strain sensor with excellent negative resistance variation and joule heating property, ACS Appl Polym Mater 5: 3338-3347. <https://doi.org/10.1021/acsapm.3c00037>.

[17] P. Ilanchezhiyan, A.S. Zakirov, G.M. Kumar, S.U. Yuldashev, H.D. Cho, T.W. Kang, A.T. Mamadalimov (2015) Highly efficient CNT functionalized cotton fabrics for flexible/wearable heating applications, RSC Advances 5: 10697-10702. <https://doi.org/10.1039/c4ra10667a>.

[18] M.S. Parvez, M.M. Rahman, M. Samykano, M. Yeakub Ali (2023) Electrochemical characterization and joule heating performance of polyaniline incorporated cotton fabric, Phys Chem Earth 129: 103323. <https://doi.org/10.1016/j.pce.2022.103323>.

[19] S. Yang, D.-X. Yan, Y. Li, J. Lei, Z.-M. Li (2021) Flexible poly(vinylidene fluoride)-MXene/silver nanowire electromagnetic shielding films with joule heating performance, Ind Eng Chem Res 60: 9824-9832. <https://doi.org/10.1021/acs.iecr.1c01632>.

[20] M.S. Sadi, E. Kumpikaitė (2023) Highly conductive composites using polypyrrole and carbon nanotubes on polydopamine functionalized cotton fabric for wearable sensing and heating applications, Cellulose 30: 7981-7999. <https://doi.org/10.1007/s10570-023-05356-9>.

[21] A. Ahmed, M.A. Jalil, M.M. Hossain, M. Moniruzzaman, B. Adak, M.T. Islam, M.S. Parvez, S. Mukhopadhyay (2020) A PEDOT:PSS and graphene-clad smart textile-based wearable electronic Joule heater with high thermal stability, J Mater Chem C 8: 16204-16215. <https://doi.org/10.1039/d0tc03368e>.

[22] P. Yotprayoonsak, N. Anusak, J. Virtanen, V. Kangas, V. Promarak (2022) Facile fabrication of flexible and conductive AuNP/DWCNT fabric with enhanced Joule heating efficiency via spray coating route, Microelectron Eng 255: 111718. <https://doi.org/10.1016/j.mee.2022.111718>.

[23] T. Alam, F. Saidane, A.a. Faisal, A. Khan, G. Hossain (2022) Smart- textile strain sensor for human joint monitoring, Sens Actuators A Phys 341: 113587. <https://doi.org/10.1016/j.sna.2022.113587>.

[24] Y. Cheng, R. Wang, J. Sun, L. Gao (2015) A Stretchable and highly sensitive graphene-based fiber for sensing tensile strain, bending, and torsion, Adv Mater 27: 7365-71. <https://doi.org/10.1002/adma.201503558>.

[25] Y. Yin, C. Guo, W. Li, H. Liu, Q. Mu (2024) A super-elastic wearable strain sensor based on PU/CNTs yarns for human-motion detection, Compos Commun 50: 102017. <https://doi.org/10.1016/j.coco.2024.102017>.

[26] S.V. Ebadi, S. Jafari (2025) Tunable electrical conductivity and enhanced strain sensing performance of MXene/polypyrrole textile sensors for wearable applications, Surf Sci 60: 106008. <https://doi.org/10.1016/j.surfin.2025.106008>.

[27] Z. Lu, J. Wang, L. He, J. Song, Z. Yang, F.A. Hammad (2024) High-performance multidirectional flexible strain sensor for human motion and health monitoring, ACS Appl Mater Interfaces 16: 41409-41420. <https://doi.org/10.1021/acsami.4c04583>.

[28] W. Zhao, Y. Zheng, J. Qian, Z. Zhaofa, Z. Jin, H. Qiu, C. Zhu, X. Hong (2022) AgNWs/MXene derived multifunctional knitted fabric capable of high electrothermal conversion efficiency, large strain and temperature sensing, and EMI shielding, J Alloys Compd 923:  166471. <https://doi.org/10.1016/j.jallcom.2022.166471>.

[29] M. Ren, J. Li, Y. Zhao, W. Zhai, K. Zhou, Y. Yu, S. Wang, K. Dai, C. Liu, C. Shen (2024) Highly strain-sensitive and stretchable multilayer conductive composite based on aligned thermoplastic polyurethane fibrous mat for human motion monitoring, Composites Communications 46: 101840. <https://doi.org/10.1016/j.coco.2024.101840>.

[30] Y. He, S. Guo, X. Zuo, M. Tian, X. Zhang, L. Qu, J. Miao (2024) Smart green cotton textiles with hierarchically responsive conductive network for personal healthcare and thermal management, ACS Appl Mater Interfaces 16: 59358-59369. <https://doi.org/10.1021/acsami.4c13999>.

[31] L. Wang, D. Wang, K. Wang, K. Jiang, G. Shen (2021) Biocompatible MXene/chitosan-based flexible bimodal devices for real-time pulse and respiratory rate monitoring, ACS Mater Lett 3: 921-929. <https://doi.org/10.1021/acsmaterialslett.1c00246>.

[32] J. Wu, Y. Yu, G. Su (2022) Safety Assessment of 2D MXenes: In Vitro and In Vivo, Nanomaterials (Basel) 12. <https://doi.org/10.3390/nano12050828>.

[33] A.M. Jastrzebska, A. Szuplewska, T. Wojciechowski, M. Chudy, W. Ziemkowska, L. Chlubny, A. Rozmyslowska, A. Olszyna (2017) In vitro studies on cytotoxicity of delaminated Ti_3_C_2_ MXene, J Hazard Mater 339: 1-8. <https://doi.org/10.1016/j.jhazmat.2017.06.004>.

[34] M. Salat, P. Petkova, J. Hoyo, I. Perelshtein, A. Gedanken, T. Tzanov (2018) Durable antimicrobial cotton textiles coated sonochemically with ZnO nanoparticles embedded in an in-situ enzymatically generated bioadhesive, Carbohydr Polym 189: 198-203. <https://doi.org/10.1016/j.carbpol.2018.02.033>.

[35] A. Verbič, M. Gorjanc, B. Simončič (2019) Zinc Oxide for functional textile coatings: recent advances, Coatings 9. <https://doi.org/10.3390/coatings9090550>.

[36] R. Rodaitė, L. Kairytė, Š.n. Varnagiris, A. Giedraitienė, R. Šiugždinienė, M. Ružauskas, I. Čiapienė, V. Tataru̅nas, D. Milčius (2025) Multifunctional WO_3_ nanoparticle-coated smart textiles via plasma sputtering: antibacterial efficiency and cellular safety, ACS Appl Nano Mater 8: 22525-22539. <https://doi.org/10.1021/acsanm.5c03018>.

[37] S.O. Ogungbesan, O. Ejeromedoghene, Y. Moglie, E. Buxaderas, B. Cui, R.A. Adedokun, M. Kalulu, M.A. Idowu, D. Díaz Díaz, G. Fu (2024) Deep eutectic solvent assisted hydrothermal synthesis of photochromic and nontoxic tungsten oxide nanoparticles, New J Chem 48: 15428-15435. <https://doi.org/10.1039/d4nj02995j>.

[38] Y. Liu, Y. Zhao, B. Sun, C. Chen (2013) Understanding the toxicity of carbon nanotubes, Acc Chem Res 46: 702-713. <https://doi.org/10.1021/ar300028m>.

[39] L. Zhou, H.J. Forman, Y. Ge, J. Lunec (2017) Multi-walled carbon nanotubes: A cytotoxicity study in relation to functionalization, dose and dispersion, Toxicol In Vitro 42: 292-298. <https://doi.org/10.1016/j.tiv.2017.04.027>.
