# Supplementary material for: Hierarchical MXene/ZnO Nanorods: WO3/CNT Trilayer Coatings on Cotton for High‐Performance Multifunctional Wearable Fabrics
Source: Adv Sci (Weinh). 2026 Mar 18;13(25):e21175. doi: 10.1002/advs.202521175 (PMC13137848; doi:10.1002/advs.202521175)
Supplement: Supplementary file 2 — Supporting File 2: advs74855‐sup‐0002‐VideoS1.pptx. [file ADVS-13-e21175-s002.pptx]

## Slide 1
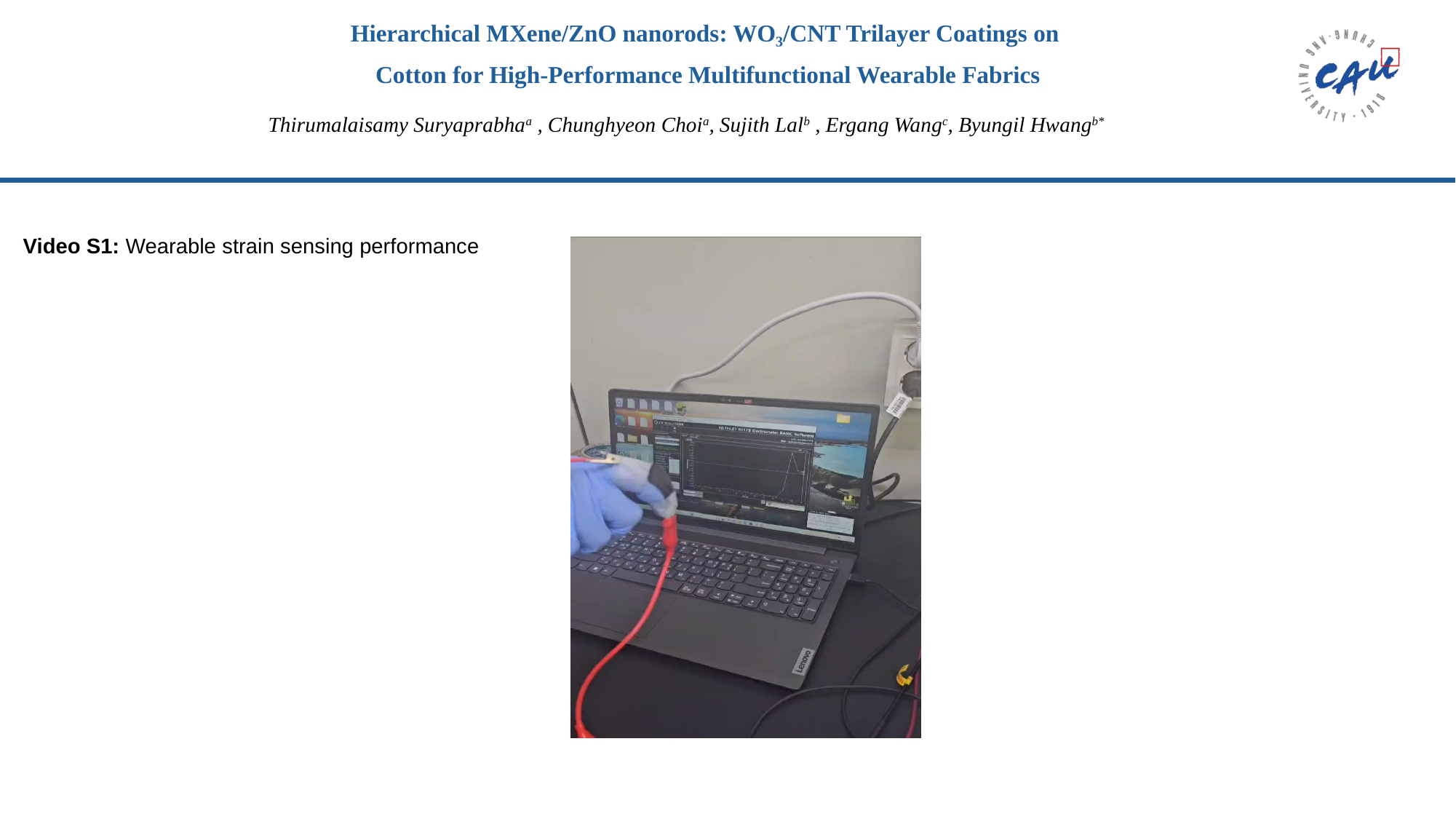

Hierarchical MXene/ZnO nanorods: WO₃/CNT Trilayer Coatings on
Cotton for High-Performance Multifunctional Wearable Fabrics
Thirumalaisamy Suryaprabhaa , Chunghyeon Choia, Sujith Lalb , Ergang Wangc, Byungil Hwangb*
Video S1: Wearable strain sensing performance

## Slide 2
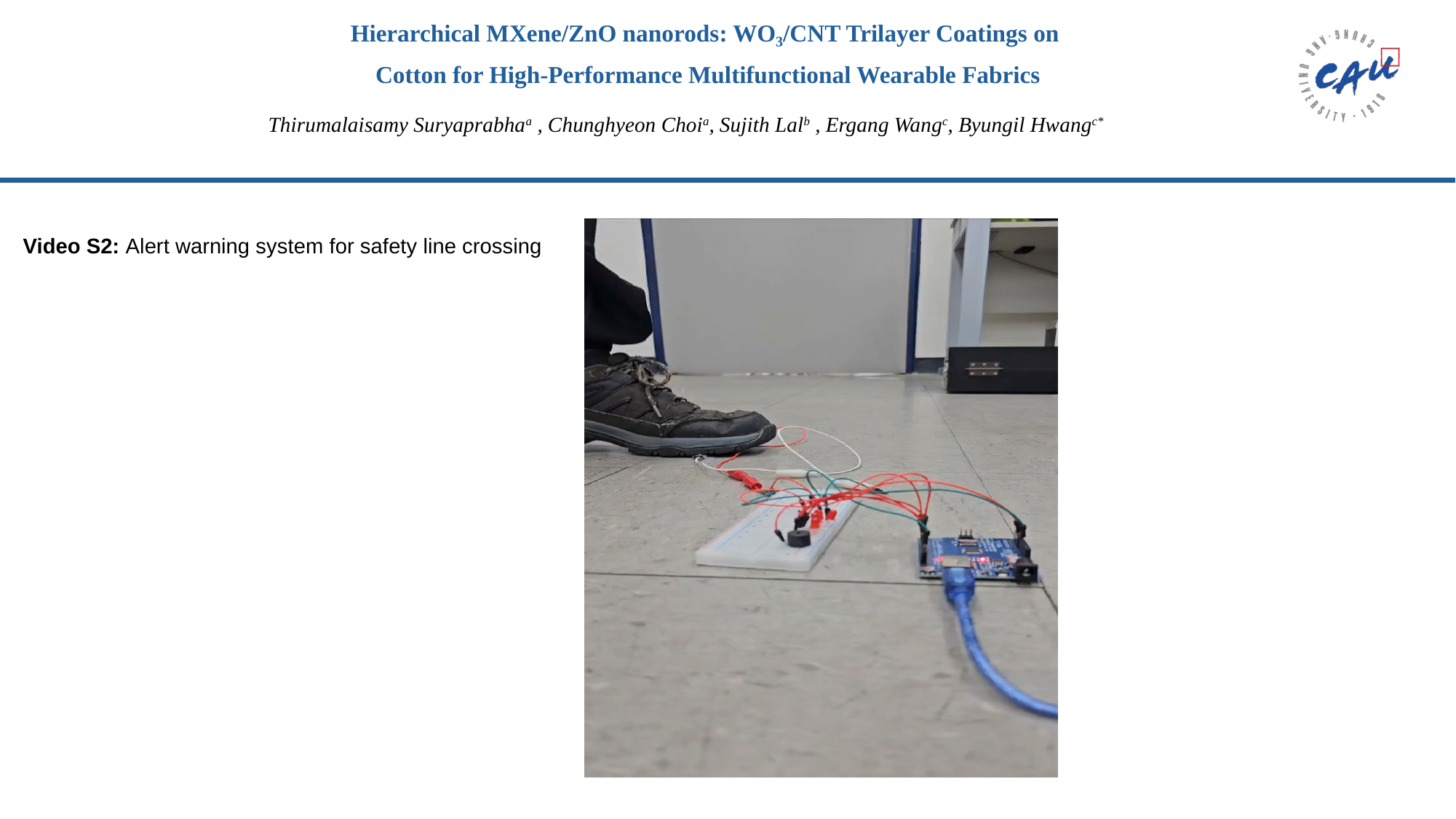

Hierarchical MXene/ZnO nanorods: WO₃/CNT Trilayer Coatings on
Cotton for High-Performance Multifunctional Wearable Fabrics
Thirumalaisamy Suryaprabhaa , Chunghyeon Choia, Sujith Lalb , Ergang Wangc, Byungil Hwangc*
Video S2: Alert warning system for safety line crossing
